# Modulation of ATXN1 S776 phosphorylation reveals the importance of allele-specific targeting in SCA1

**DOI:** 10.1172/jci.insight.144955

**Published:** 2021-02-08

**Authors:** Larissa Nitschke, Stephanie L. Coffin, Eder Xhako, Dany B. El-Najjar, James P. Orengo, Elizabeth Alcala, Yanwan Dai, Ying-Wooi Wan, Zhandong Liu, Harry T. Orr, Huda Y. Zoghbi

**Affiliations:** 1 Program in Integrative Molecular and Biomedical Sciences and; 2 Department of Molecular and Human Genetics, Baylor College of Medicine, Houston, Texas, USA.; 3 Jan and Dan Duncan Neurological Research Institute at Texas Children’s Hospital, Houston, Texas, USA.; 4 Program in Genetics and Genomics,; 5 Department of Neurology, and; 6 Department of Pediatrics, Baylor College of Medicine, Houston, Texas, USA.; 7 Institute for Translational Neuroscience and Department of Laboratory Medicine and Pathology, University of Minnesota Medical School, Minneapolis, Minnesota, USA.; 8 Howard Hughes Medical Institute, Houston, Texas, USA.

**Keywords:** Genetics, Neuroscience, Genetic diseases, Mouse models, Neurodegeneration

## Abstract

Spinocerebellar ataxia type 1 (SCA1) is an adult-onset neurodegenerative disorder characterized by motor incoordination, mild cognitive decline, respiratory dysfunction, and early lethality. It is caused by the expansion of the polyglutamine (polyQ) tract in Ataxin-1 (ATXN1), which stabilizes the protein, leading to its toxic accumulation in neurons. Previously, we showed that serine 776 (S776) phosphorylation is critical for ATXN1 stability and contributes to its toxicity in cerebellar Purkinje cells. Still, the therapeutic potential of disrupting S776 phosphorylation on noncerebellar SCA1 phenotypes remains unstudied. Here, we report that abolishing S776 phosphorylation specifically on the polyQ-expanded ATXN1 of SCA1-knockin mice reduces ATXN1 throughout the brain and not only rescues the cerebellar motor incoordination but also improves respiratory function and extends survival while not affecting the hippocampal learning and memory deficits. As therapeutic approaches are likely to decrease S776 phosphorylation on polyQ-expanded and WT ATXN1, we further disrupted S776 phosphorylation on both alleles and observed an attenuated rescue, demonstrating a potential protective role of WT allele. This study not only highlights the role of S776 phosphorylation to regulate ATXN1 levels throughout the brain but also suggests distinct brain region–specific disease mechanisms and demonstrates the importance of developing allele-specific therapies for maximal benefits in SCA1.

## Introduction

Spinocerebellar ataxia type 1 (SCA1) is a dominantly inherited neurodegenerative disease caused by the expansion of CAG repeats encoding the polyglutamine (polyQ) tract in Ataxin-1 (ATXN1) (1, 2). The disease typically manifests in adulthood and is characterized by motor incoordination, mild cognitive decline, respiratory dysfunction, and, consequently, death (3, 4). These symptoms are thought to be attributed to the progressive degeneration of neurons in multiple brain regions, including (a) the cerebellum, which is critical for the coordination of movements; (b) the hippocampus, which is in charge of the formation and consolidation of memories; and (c) the brainstem, which controls essential functions such as the coordination of breathing and swallowing (3, 5, 6). To date, no treatment options are available to cure or halt the progression of SCA1.

Previous studies showed that the polyQ-expansion stabilizes ATXN1, leading to its toxic accumulation in the nucleus of affected neurons (7, 8). Attempts to decrease ATXN1 levels have since been shown to mitigate the cerebellar SCA1 phenotypes in mice (9–12). One strategy to lower ATXN1 levels is by modulating the phosphorylation of serine 776 (S776) in ATXN1 (9, 13). S776 is phosphorylated via kinases such as MSK1 and PKA (13, 14). The phosphorylation event stabilizes ATXN1 by recruiting 14-3-3, a protein that binds and protects ATXN1 from degradation (15–17). Disruption of S776 phosphorylation in a transgenic SCA1 mouse model overexpressing human ATXN1[82Q] exclusively in cerebellar Purkinje cells (*PCP2-hATXN1[82Q]*) reduced hATXN1[82Q] transgene protein levels and improved the cerebellar motor coordination phenotypes (9). This seminal work demonstrated that disruption of S776 phosphorylation might serve as a potential therapeutic entry point for SCA1.

Still, targeting S776 phosphorylation as a therapeutic approach for SCA1 remains an uncertain possibility, as, thus far, no studies have investigated the effect of abolishing endogenous S776 phosphorylation on ATXN1 levels and SCA1 pathogenesis outside the cerebellum. It is important to note that therapeutic strategies, such as kinase inhibitors, would reduce S776 phosphorylation on both the polyQ-expanded and WT ATXN1. Loss of WT *Atxn1* was previously shown to worsen SCA1 motor coordination and survival phenotypes (18); therefore, the question remains if disrupting phosphorylation on both alleles will be safe and effective.

Here, we show that ATXN1 S776 is phosphorylated throughout the brain. We use CRISPR/Cas9 technology to disrupt endogenous S776 phosphorylation, by mutating the serine to alanine (S776A), on the unexpanded WT *Atxn1[2Q]* allele of C57BL/6J WT animals, and on the polyQ-expanded *Atxn1[154Q]* allele of an existing SCA1-knockin mouse model (*Atxn1^154Q/2Q^*), which recapitulates all major human SCA1 features (19). We find that independent disruption of S776 phosphorylation on either the WT or polyQ-expanded ATXN1 reduces its protein levels throughout all affected brain regions. Disruption of S776 phosphorylation specifically on the polyQ-expanded ATXN1 not only improves the motor coordination but also partially ameliorates the neuromuscular respiratory dysfunction and extends the life span, while having no effect on the hippocampal learning and memory deficits of SCA1 animals. Interestingly, we show that SCA1 animals with homozygous S776A mutations (on both the *Atxn1[154Q]* and *Atxn1[2Q]* allele) displayed a reduced rescue compared with mice with the S776A mutation on only the expanded allele, suggesting that the WT ATXN1 might exert neuroprotective properties.

These findings highlight the potential of disrupting S776 phosphorylation as a means for reducing ATXN1 levels throughout the brain, suggest distinct brain region–specific disease mechanisms, and show that the polyQ-expanded *ATXN1* allele should be selectively targeted for maximal therapeutic benefit.

## Results

### ATXN1 S776 is phosphorylated in various tissues.

**Figure 1:**
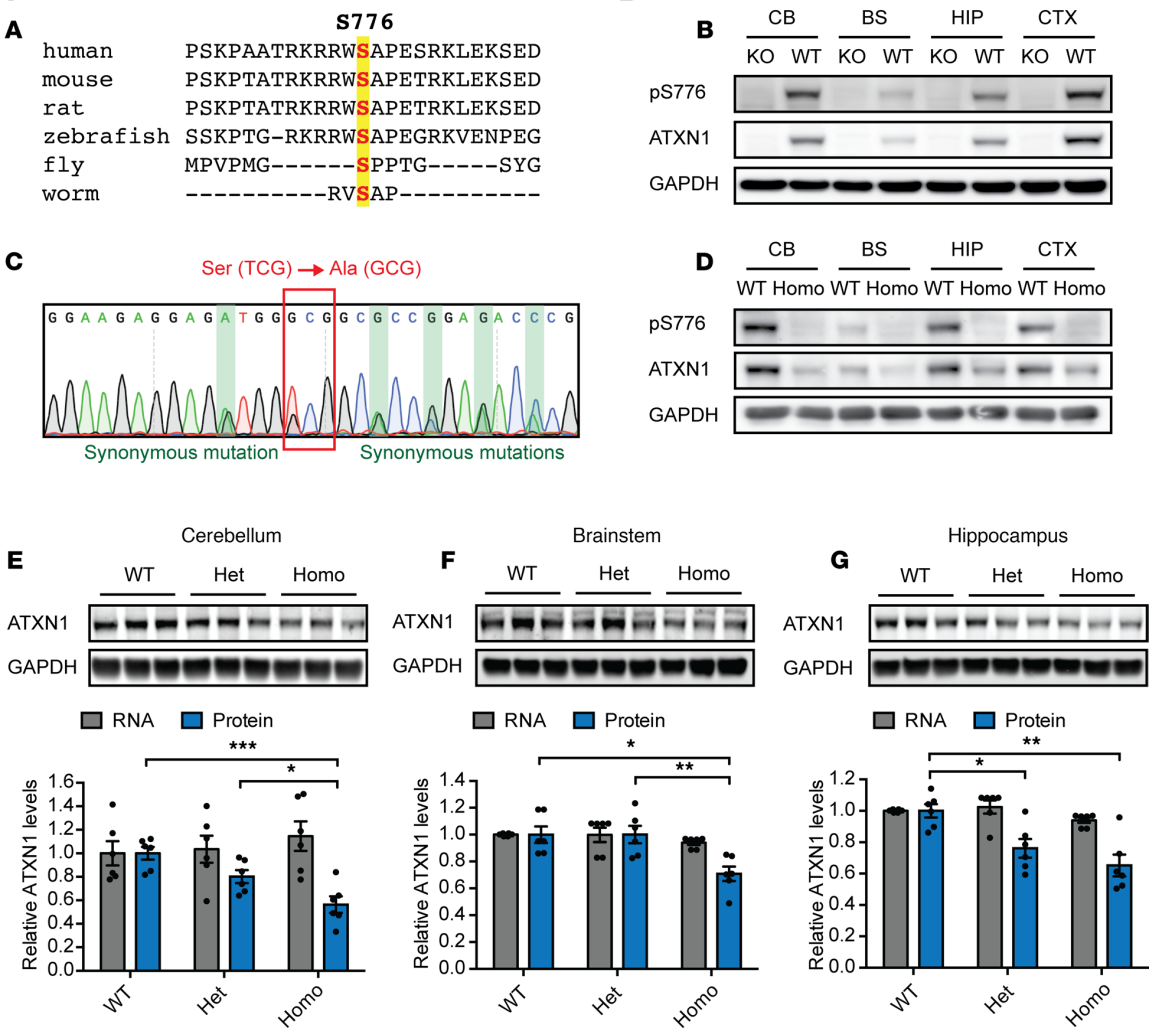
Disruption of S776 phosphorylation reduces ATXN1[2Q] levels in the cerebellum, brainstem, and hippocampus. (**A**) Conservation of ATXN1 S776 across multiple species. (**B**) Representative Western blot showing ATXN1 S776 phosphorylation in the cerebellum (CB), brainstem (BS), hippocampus (HIP), and cortex (CTX) of 6-week-old *Atxn1^–/–^* (KO) and *Atxn1^2Q/2Q^* (WT) mice. (**C**) Sanger sequencing confirming the serine-to-alanine mutation at position 776 and synonymous mutations in heterozygous *Atxn1^2Q[S776A]/2Q^* F1 offspring upon CRISPR injections. (**D**) Representative Western blot confirming loss of S776 phosphorylation in 6-week-old *Atxn1^2Q[S776A]/2Q[S776A]^* (Homo) mice. Representative Western blot and quantifications of *Atxn1* RNA and protein levels in the (**E**) cerebellum, (**F**) brainstem, and (**G**) hippocampus of 6-week-old *Atxn1^2Q/2Q^* (WT), *Atxn1^2Q[S776A]/2Q^* (Het), and *Atxn1^2Q[S776A]/2Q[S776A]^* (Homo) mice. For each assay, a minimum of 3 replicates were performed. Multigroup comparisons used 1-way ANOVAs. *P *<* 0.05, **P *<* 0.01, ***P *<* 0.001. All data represent means ± SEM.

ATXN1 S776 is highly conserved among species (Figure 1A) and was shown to be phosphorylated in mice and humans (9, 16). Previous studies showed that disruption of S776 phosphorylation in the transgenic *PCP2-hATXN1[82Q]* SCA1 mouse model reduces hATXN1[82Q] protein levels in the cerebellum (9). Still, as the hATXN1[82Q] protein was only expressed in cerebellar Purkinje cells, it remains unclear in which other tissues S776 is phosphorylated and if its disruption could reduce ATXN1 levels in all brain regions affected in SCA1.

To address these questions, we first used an ATXN1 S776 phospho-specific antibody on protein extracts of C57BL/6J WT mice (9). Although *Atxn1^–/–^* KO controls showed no antibody signal, S776 phosphorylation was detected in all tested brain regions, including the cerebellum, brainstem, hippocampus, and cortex (Figure 1B), as well as all tested peripheral tissues, including the heart, lung, spleen, and muscle of WT mice (Supplemental Figure 1A; supplemental material available online with this article; https://doi.org/10.1172/jci.insight.144955DS1). These data show that endogenous ATXN1 is phosphorylated at S776 in various tissues throughout the mouse, suggesting that S776 phosphorylation may regulate ATXN1 levels in cerebellar Purkinje cells and throughout the brain and peripheral tissues.

### S776 phosphorylation stabilizes ATXN1 levels throughout the brain.

To investigate whether loss of endogenous S776 phosphorylation decreases ATXN1 levels in the cerebellum and other brain regions affected in SCA1, we ablated endogenous S776 phosphorylation in vivo. Using CRISPR/Cas9 technology, we introduced a nonphosphorylatable serine-to-alanine mutation at position 776 (S776A) on the unexpanded *Atxn1[2Q]* allele of C57BL/6J WT mice to generate *Atxn1^2Q[S776A]/2Q^* animals. Additional synonymous mutations were added to facilitate genotyping. Sanger sequencing confirmed the correct mutations in the F1 heterozygous offspring of 2 lines (Figure 1C and Supplemental Figure 1B).

After backcrossing, we bred *Atxn1^2Q/2Q^* (WT), *Atxn1^2Q[S776A]/2Q^* (WT S776A heterozygous), and *Atxn1^2Q[S776A]/2Q[S776A]^* (WT S776A homozygous) mice (Supplemental Figure 1C). Specific primers for the WT and S776A allele were used to distinguish genotypes (Supplemental Figure 1D). We confirmed the absence of S776 phosphorylation in WT S776A homozygous animals by Western blotting (Figure 1D and Supplemental Figure 1E), thus validating the phospho-mutant effect of the serine-to-alanine mutation on S776 phosphorylation. To ensure that the added mutations did not impact *Atxn1* mRNA stability, we extracted RNA from the 3 brain regions known to be affected in SCA1: the cerebellum, brainstem, and hippocampus. No differences in *Atxn1* mRNA levels were found between the genotypes by quantitative real-time PCR (RT-qPCR, Figure 1, E–G; gray bars). Importantly, we also performed Western blotting to measure if disruption of S776 phosphorylation could indeed reduce ATXN1 protein levels in the affected brain regions. We found that ATXN1[2Q] protein levels were reduced in all 3 brain regions in the WT S776A homozygous mice (Figure 1, E–G; blue bars), indicating a universal effect of S776 phosphorylation on ATXN1 stability throughout the brain.

To ensure that this effect was not unique to the progeny of 1 founder, we repeated the RT-qPCR and Western blot analysis on samples from the offspring of the second founder. Again, we found that whereas *Atxn1* mRNA levels were unchanged (Supplemental Figure 1, F–H; gray bars), ATXN1[2Q] protein levels were decreased in all brain regions of the S776A homozygous mice (Supplemental Figure 1, F–H; blue bars).

These data show that S776 phosphorylation stabilizes endogenous WT ATXN1 in the cerebellum and other brain regions affected in SCA1, raising the question whether polyQ-expanded ATXN1 is regulated in a similar fashion.

### Disruption of S776 phosphorylation reduces polyQ-expanded ATXN1.

**Figure 2:**
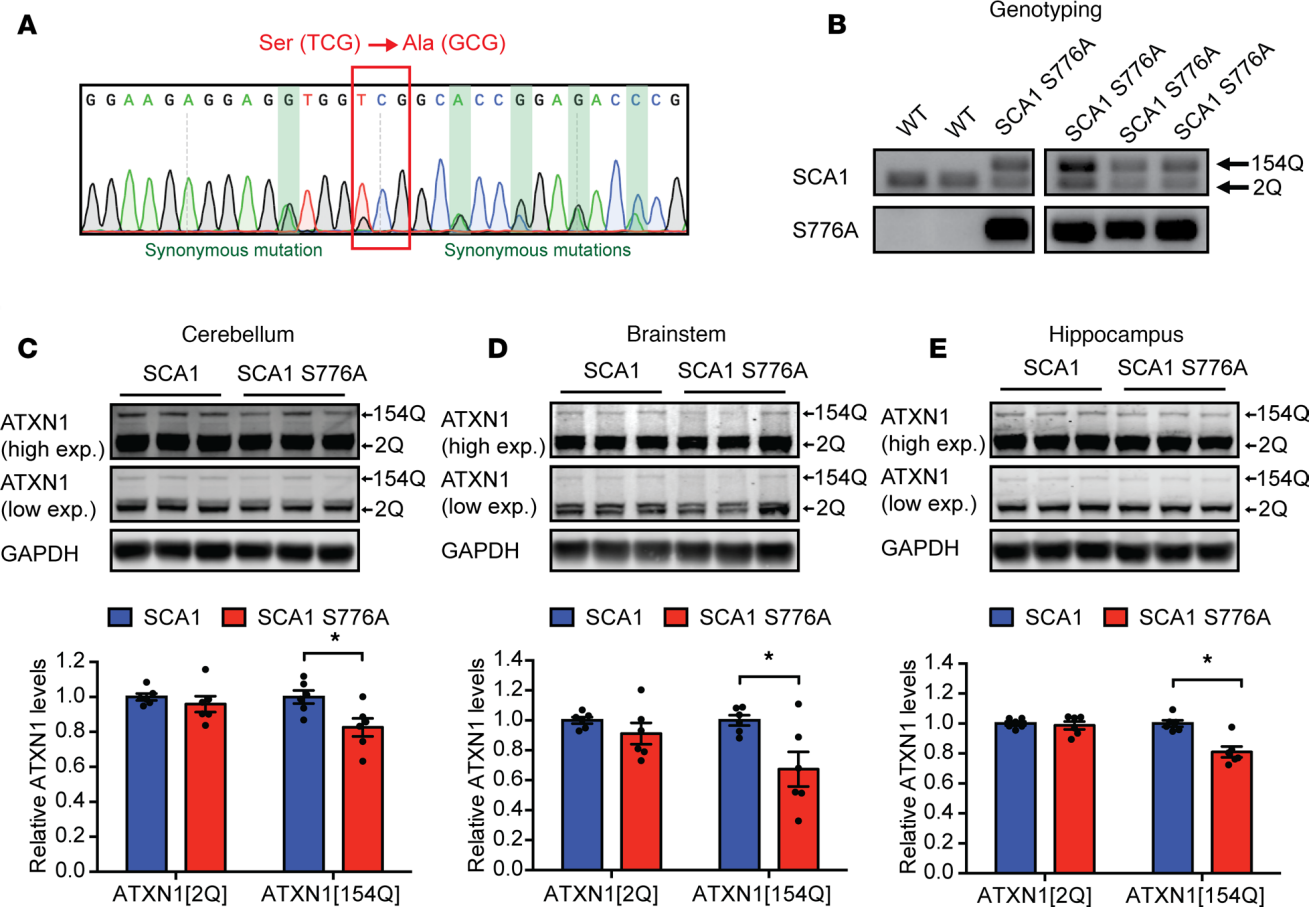
Disruption of S776 phosphorylation reduces polyQ-expanded ATXN1[154Q] levels in the cerebellum, brainstem, and hippocampus. (**A**) Sanger sequencing confirming serine-to-alanine mutation at position 776 and synonymous mutations in heterozygous *Atxn1^154Q[S776A]/2Q^* F1 offspring upon CRISPR injections. (**B**) Genotyping of F1 offspring using specific primers to distinguish WT and SCA1 mice as well as primers for the detection of the S776A allele. Representative Western blots and quantifications of ATXN1[2Q] and ATXN1[154Q] protein levels in the (**C**) cerebellum, (**D**) brainstem, and (**E**) hippocampus of 6-week-old *Atxn1^154Q/2Q^* (SCA1) and *Atxn1^154Q[S776A]/2Q^* (SCA1 S776A) mice. For each assay, a minimum of 6 replicates were performed. Simple comparisons used Student’s *t* test. *P *<* 0.05. All data represent means ± SEM.

Given that disruption of S776 phosphorylation decreased unexpanded ATXN1[2Q] levels throughout the brain, we next sought to investigate if abolishing S776 phosphorylation in polyQ-expanded ATXN1 would have a similar effect. To this end, we used CRISPR/Cas9 to edit S776 in an existing *Atxn1^154Q/2Q^* (SCA1) knockin mouse model, which, similar to SCA1 patients, expresses 1 *Atxn1* allele with a CAG-repeat expansion (*Atxn1[154Q]*) and 1 unexpanded WT *Atxn1* (*Atxn1[2Q]*) allele (19). We confirmed the insertion of the correct mutation by Sanger sequencing (Figure 2A and Supplemental Figure 2A). The segregation of the *Atxn1[154Q]* allele with S776A genotyping confirmed the correct location of the serine-to-alanine mutation on the *Atxn1[154Q]* allele in 2 separate *Atxn1^154Q[S776A]/2Q^* (SCA1 S776A) lines (Figure 2B and Supplemental Figure 2B).

After backcrossing, we again measured ATXN1 protein levels in the cerebellum, brainstem, and hippocampus. Disruption of S776 phosphorylation in the polyQ-expanded ATXN1 reduced the protein levels of ATXN1[154Q] in all tested brain regions in both founder lines (Figure 2, C–E; and Supplemental Figure 2, C–E). In contrast, ATXN1[2Q] protein levels were unchanged (Figure 2, C–E; and Supplemental Figure 2, C–E). These data show that disruption of S776 phosphorylation could indeed reduce polyQ-expanded ATXN1 in all brain regions affected in SCA1 and led us to explore whether its disruption could improve the cerebellar motor coordination deficits as well as the noncerebellar SCA1 phenotypes.

### Disruption of S776 phosphorylation improves cerebellar SCA1 pathogenesis.

SCA1 mice recapitulate many pathological and behavioral characteristics of patients affected with SCA1 and develop first symptoms of SCA1 around 5 weeks of age (6, 19). To determine whether lower ATXN1 levels resulting from loss of S776 phosphorylation mitigate behavioral deficits in SCA1 mice, we performed a battery of behavioral tests on WT, SCA1, and SCA1 S776A mice.

**Figure 3:**
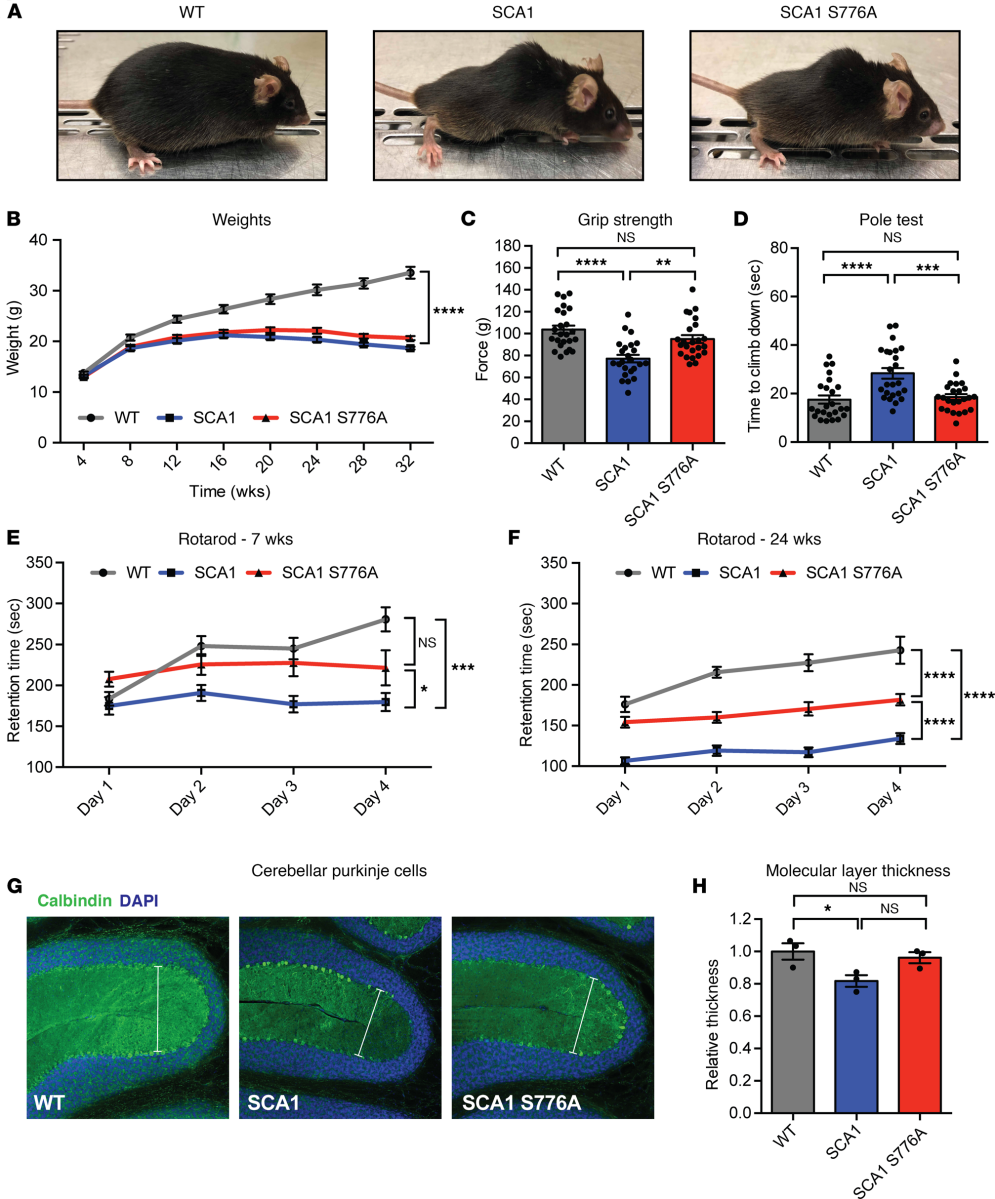
Disruption of S776 phosphorylation improves SCA1 motor coordination deficits and cerebellar Purkinje cell degeneration. (**A**) Pictures of *Atxn1^2Q/2Q^* (WT), *Atxn1^154Q/2Q^* (SCA1) and *Atxn1^154Q[S776A]/2Q^* (SCA1 S776A) mice at 40 weeks of age. (**B**) Monthly weights. (**C**) Grip strength test and (**D**) pole test at 7 weeks of age. (**E**) Rotarod test at 7 and (**F**) 24 weeks of age. For each behavioral assay 24 mice per genotype were used. (**G**) Cerebellar Purkinje cells stained with DAPI (blue) and Calbindin (green) at 44 weeks of age. Original magnification, ×20. (**H**) Quantification of the molecular layer thickness in the cerebellum of 3 mice per genotype. Multigroup comparisons used 1- and 2-way ANOVAs. *P *<* 0.05, **P *<* 0.01, ***P *<* 0.001, ****P *<* 0.0001, NS, *P* *>* 0.05. All data represent means ± SEM.

Two of the most visually striking features of SCA1 mice, the formation of a hunched posture (kyphosis) and the failure to gain weight, remained unaltered in the SCA1 S776A mice (Figure 3, A and B). SCA1 mice also develop motor incoordination, a major feature seen in individuals with SCA1, and have reduced muscle strength (19, 20). At 7 weeks of age, loss of S776 phosphorylation in SCA1 mice resulted in a complete rescue of the grip strength phenotype (Figure 3C). In addition, motor coordination deficits, measured by the pole and rotarod tests, were also rescued at the early time point (Figure 3, D and E). To investigate if this rescue was maintained as the mice aged, we repeated the rotarod assay at 24 weeks of age (Figure 3F). As expected, motor coordination deteriorated in SCA1 mice at 24 weeks of age but was partially restored in SCA1 S776A mice (Figure 3F), suggesting a partial rescue of the motor coordination phenotypes.

Impaired motor coordination in SCA1 is caused by the degeneration of cerebellar Purkinje cells (19). Therefore, to determine whether the improved motor phenotype observed in SCA1 S776A mice corresponds with improved health of cerebellar Purkinje cells, we performed immunostaining for Calbindin, a calcium-binding protein that is highly expressed in Purkinje cells, on cerebella from 44-week-old animals (Figure 3G). In accordance with previous findings (21), we found that the thickness of the cerebellar molecular layer was reduced in SCA1 animals, indicating significant dendritic loss. However, this was rescued in SCA1 S776A mice, which had a cerebellar molecular layer thickness comparable to WT mice (Figure 3H).

PolyQ-expanded ATXN1 tends to form nuclear inclusions in cerebellar Purkinje cells (19). Disruption of S776 phosphorylation in the transgenic *PCP2-hATXN1[82Q]* mouse model was previously shown to abolish ATXN1 inclusion formation (9). Therefore, we stained the cerebellar cortex with anti-ATXN1 11NQ antibody to determine if inclusion formation is abolished in the SCA1 S776A mice (Supplemental Figure 3A). Consistent with previous work (9, 22), at 37 weeks of age, SCA1 mice displayed nuclear inclusions in about 50% of cerebellar Purkinje cells; in contrast no inclusion was found in the SCA1 S776A mice (Supplemental Figure 3B).

Altogether, these data demonstrate that disruption of S776 phosphorylation improved the muscle strength and motor coordination phenotypes in the SCA1-knockin mouse model but did not affect weight loss or kyphosis. The partial rescue of motor coordination is likely attributed to an improvement of the cerebellar Purkinje cell dysfunction and suppression of the pathology.

### Abolishing S776 phosphorylation has no impact on hippocampal phenotypes.

Some individuals with SCA1 experience mild cognitive impairment (4). Similarly, SCA1 mice develop learning and memory deficits (19). To test whether the loss of S776 phosphorylation in SCA1 mice can rescue the learning and memory deficits, we performed the Morris water maze, a hippocampus-dependent memory task in which mice use spatial cues to find a hidden platform in a circular pool of opaque water, at 14 weeks of age (Supplemental Figure 4A). Mice with learning and memory deficits take longer to find the platform during a 4-day trial period and fail to remember the location of the platform during the probe trial when the platform was removed (Supplemental Figure 4A). On day 1, SCA1 and SCA1 S776A mice displayed similar swim speed to WT mice (Supplemental Figure 4B). Notably, both SCA1 and SCA1 S776A animals took a longer time than WT mice to find the hidden platform over the 4-day period (Supplemental Figure 4C). Similarly, during the probe trial, both SCA1 and SCA1 S776A animals failed to remember the platform location (Supplemental Figure 4, D and E), suggesting that disruption of S776 phosphorylation and the resulting reduction of the polyQ-expanded ATXN1 levels did not rescue the learning and memory deficits.

As SCA1 mice displayed notable motor impairment by 14 weeks of age, a concern was that the poor performance of SCA1 and SCA1 S776A mice in the Morris water maze was attributed to their inability to swim. To assess the competency of SCA1 and SCA1 S776A mice to complete the Morris water maze, we performed the visible platform test on another cohort of mice at the same age. We again found no difference in the swim speed of WT, SCA1, and SCA1 S776A mice on day 1 (Supplemental Figure 4F), suggesting normal motor performance. Moreover, both SCA1 and SCA1 S776A mice performed similarly to WT mice throughout the 4-day trial period (Supplemental Figure 4G). This indicates that the poor performance of SCA1 and SCA1 S776A mice in the Morris water maze is attributed to learning and memory deficits, and that disruption of S776 phosphorylation does not improve the learning and memory deficits.

### Delay of breathing dysfunction and lethality in SCA1 S776A mice.

**Figure 4:**
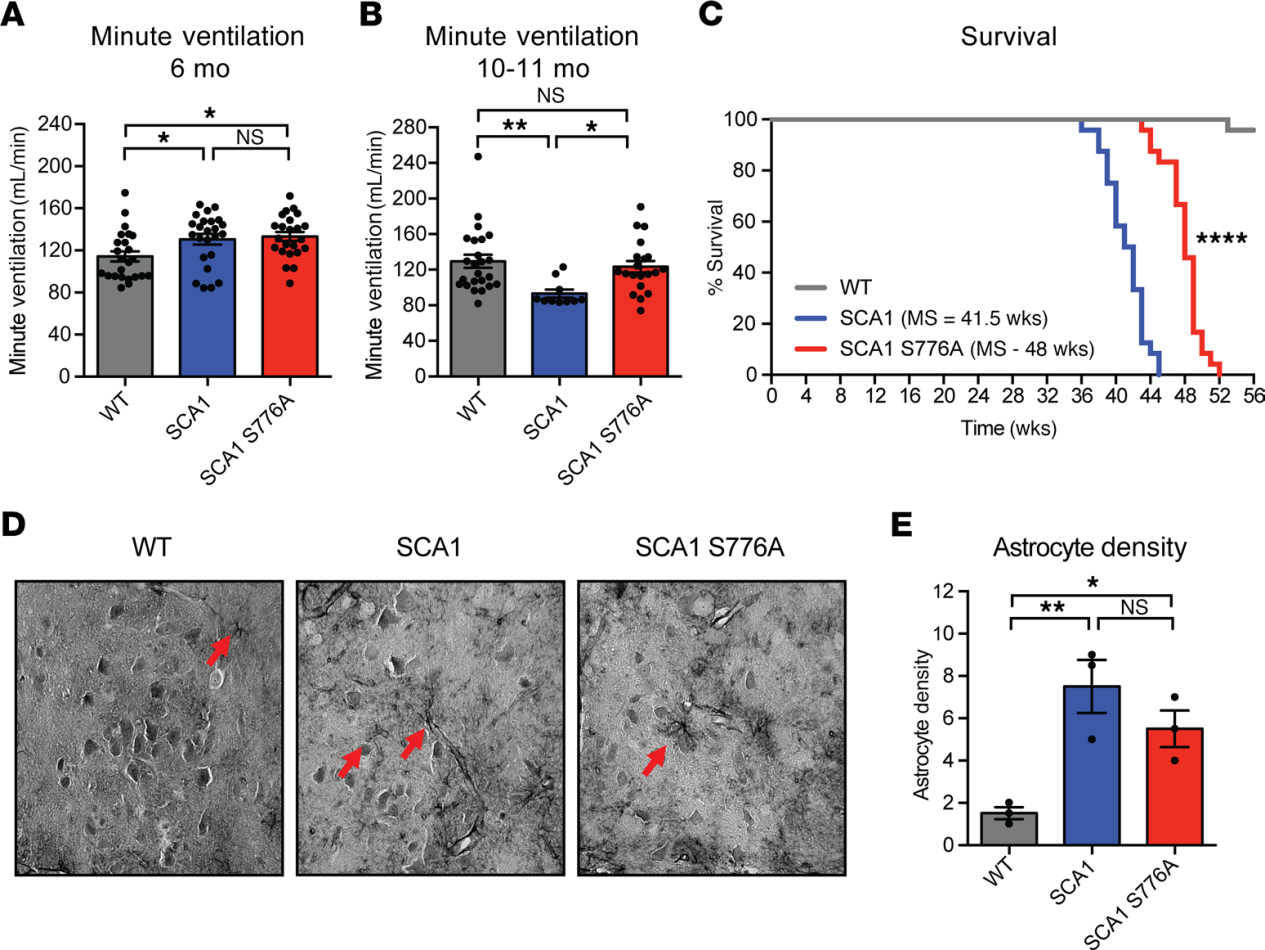
Disruption of S776 phosphorylation improves SCA1 breathing dysfunction and extends survival. Minute ventilation of *Atxn1^2Q/2Q^* (WT), *Atxn1^154Q/2Q^* (SCA1), and *Atxn1^154Q[S776A]/2Q^* (SCA 1S776A) mice at (**A**) 6 months and (**B**) 10–11 months of age. A minimum of 10 animals per genotype were used. (**C**) Survival analysis and median survival (MS) of 24 animals per genotype. (**D**) Representative GFAP immunostainings performed to identify astrocytes in the hypoglossal nucleus in paraffin-embedded tissue from 30-week-old mice. (**E**) Quantification of the astrocyte density (number of astrocytes per region of interest, 90,000 μm^2^) in the brainstem of 3 mice per genotype. Multigroup comparisons used 1-way ANOVAs. Survival analysis used log-rank test. *P *<* 0.05, **P *<* 0.01, ****P *<* 0.0001, NS, *P* *>* 0.05. All data represent means ± SEM.

SCA1 individuals typically pass away from complications of bulbar dysfunction 10–20 years after disease onset (3). Similarly, SCA1 mice develop neuromuscular breathing dysfunction at 6 months of age and die prematurely (6, 19). To assess whether disrupting ATXN1 S776 phosphorylation could reverse these phenotypes, we performed whole-body plethysmography to measure breathing in WT, SCA1, and SCA1 S776A mice at 6 months of age. Similar to previous reports (6), SCA1 animals showed a decrease in tidal volume with a compensatory increase in breathing frequency compared with controls (Supplemental Figure 5, A and B). SCA1 S776A mice performed comparably to SCA1 at this time point (Supplemental Figure 5, A and B). Owing to the compensatory increase in breathing frequency, mice of both genotypes displayed a mild increase in minute ventilation, the volume inhaled per minute (Figure 4A). Interestingly, when we repeated whole-body plethysmography at 10–11 months of age, close to the end of life for SCA1 animals, we found that SCA1 animals had a more pronounced decrease in tidal volume but were unable to further increase their breathing frequency (Supplemental Figure 5, C and D). This resulted in a significant reduction in their minute ventilation (Figure 4B). In contrast, SCA1 S776A mice displayed a slight improvement in the tidal volume (not statistically different) and a significant increase in the breathing frequency compared with SCA1 mice, resulting in an improvement of their minute ventilation (Supplemental Figure 5, C and D; and Figure 4B).

Because breathing dysfunction is a determining factor for the survival of SCA1 mice, we next assessed their life span. We found that SCA1 animals had a median survival of 41.5 weeks, whereas SCA1 S776A mice lived 6–7 weeks longer with a median survival of 48 weeks (Figure 4C).

Our laboratory has previously demonstrated that the breathing dysfunction seen in SCA1 mice correlates with the degeneration of motor neurons and increased astrocytosis in the brainstem and cervical spinal cord (6). Therefore, we also investigated whether a decrease in motor neuron degeneration could explain the delay in breathing dysfunction and extension of survival. In 30-week-old WT, SCA1, and SCA1 S776A mice, we surveyed the hypoglossal nucleus in the brainstem with hematoxylin and eosin staining to look at motor neuron morphology. At this point we did not find a difference in the number or morphology of the motor neurons between the 3 groups (data not shown). However, using glial fibrillary astrocyte protein (GFAP) immunostaining on the same area in the brainstem, we did find significantly increased reactive astrocytosis in SCA1 mice (Figure 4, D and E). In contrast, SCA1 S776A mice showed a slight, but not statistically different, reduction in astrocyte density compared with SCA1 mice (Figure 4, D and E). These data show that disruption of S776 phosphorylation on the polyQ-expanded ATXN1 delays neuromuscular breathing failure and extends survival.

### Reduction of phosphorylation on both alleles decreases the rescue effect.

**Figure 5:**
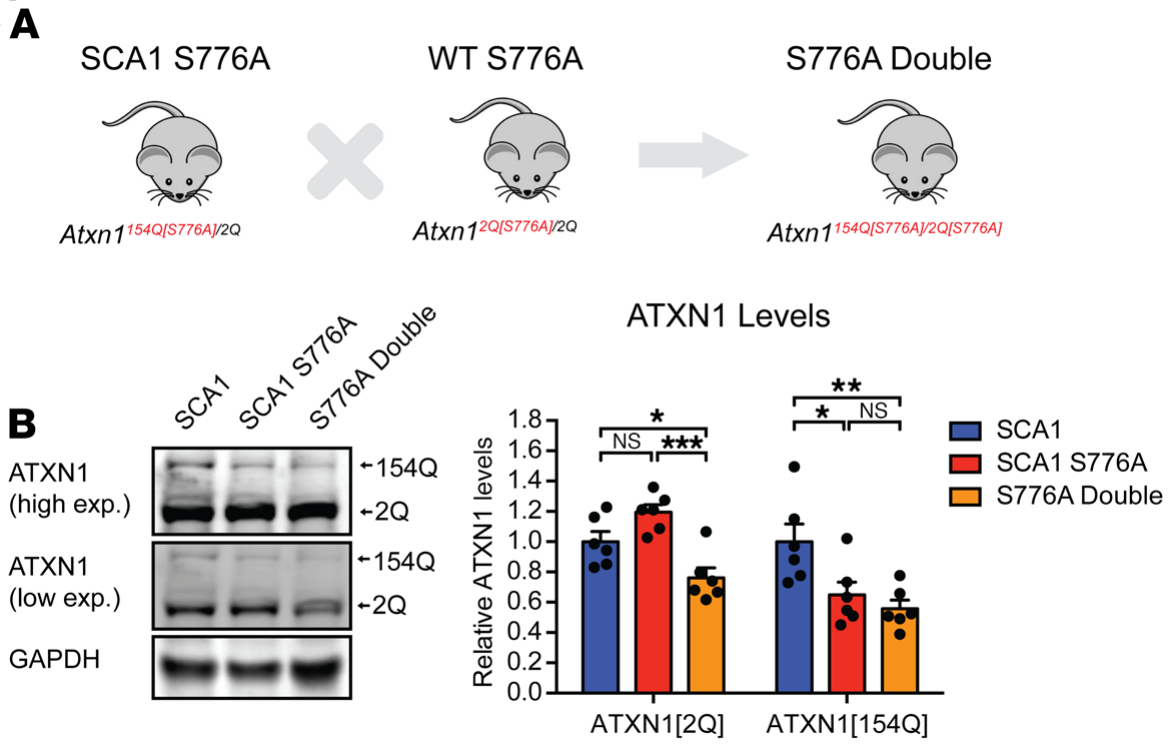
S776A double mutants have reduced ATXN1[2Q] and ATXN1[154Q] levels. (**A**) Breeding scheme to generate *Atxn1^154Q[S776A]/2Q[S776A]^* (S776A double mutants). (**B**) Representative Western blot and quantification of ATXN1[2Q] and ATXN1[154Q] levels in 4-week-old *Atxn1^154Q/2Q^* (SCA1), *Atxn1^154Q[S776A]/2Q^* (SCA1 S776A), and *Atxn1^154Q[S776A]/2Q[S776A]^* (S776A double mutants). A minimum of 6 replicates were performed. Multigroup comparisons used 1-way ANOVAs. *P *<* 0.05, **P *<* 0.01, ***P *<* 0.001, NS, *P* *>* 0.05. All data represent means ± SEM.

Although we found an improvement of the SCA1 motor incoordination, a delay in respiratory failure, and an extension in life span in the SCA1 S776A mice, therapeutic approaches likely would decrease S776 phosphorylation on both the polyQ-expanded and unexpanded WT ATXN1. To test if there is a difference in the therapeutic efficacy of reducing polyQ-expanded ATXN1 alone or both the polyQ-expanded and unexpanded allele in combination, we crossed WT S776A heterozygous and SCA1 S776A mice to generate *Atxn1^154Q[S776A]/2Q[S776A]^* (S776A double mutants) mice (Figure 5A). Western blot analysis revealed that ATXN1[154Q] was decreased to a similar extent in the SCA1 S776A and S776A double mutant mice, whereas ATXN1[2Q] was only reduced in the S776A double mutants (Figure 5B).

**Figure 6:**
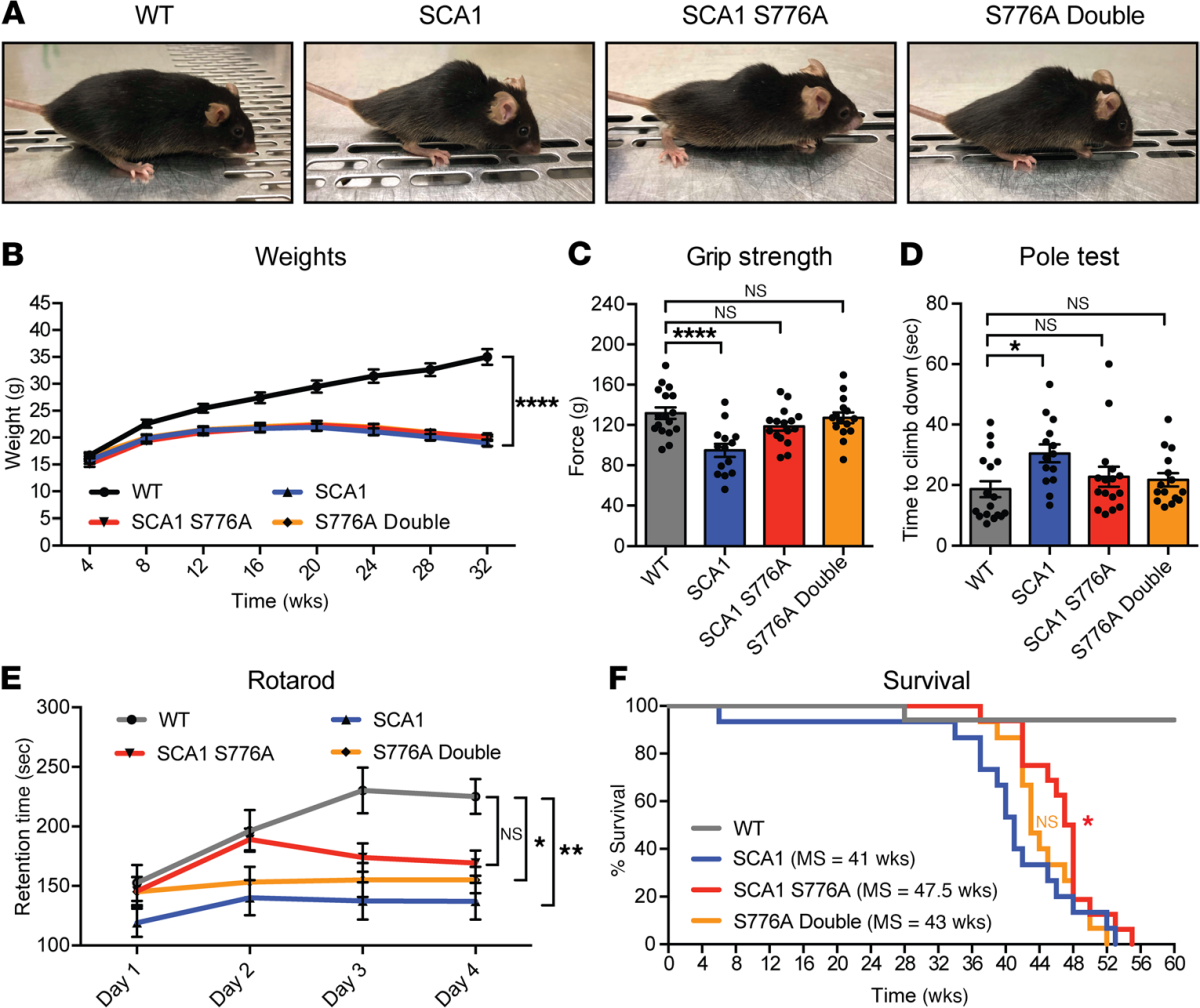
Disruption of S776 phosphorylation on both alleles reduces the behavioral rescue. (**A**) Pictures of *Atxn1^2Q/2Q^* (WT), *Atxn1^154Q/2Q^* (SCA1), and *Atxn1^154Q[S776A]/2Q^* (SCA1 S776A) mice and *Atxn1^154Q[S776A]/2Q[S776A]^* (S776A double) at 40 weeks of age. (**B**) Monthly weights. (**C**) Grip strength test and (**D**) pole test at 7 weeks of age. (**E**) Rotarod test at 24 weeks of age. (**F**) Survival analysis and median survival (MS). For each behavioral assay a minimum of 14 animals per genotype were used. Multigroup comparisons used 1- and 2-way ANOVAs. Survival analysis used log-rank test. *P *<* 0.05, **P *<* 0.01, ****P *<* 0.0001, NS, *P* *>* 0.05. All data represent means ± SEM.

Similar to SCA1 and SCA1 S776A mice, S776A double mutants develop kyphosis (Figure 6A) and fail to gain weight (Figure 6B). Therefore, we next wanted to see whether disruption of S776 phosphorylation on both alleles of the SCA1 mouse could affect muscle strength and motor coordination deficits and repeated the grip strength and pole tests, as well as the rotarod assays. In the grip strength and pole test assays, S776A double mutants exhibited a rescue equal in size to that seen in single SCA1 S776A mutant mice (Figure 6, C and D). Interestingly, the rescue was less pronounced in the rotarod test (Figure 6E), suggesting a protective effect of unexpanded ATXN1 in regard to the motor coordination phenotypes in SCA1.

We observed a similar effect in regard to the life span of S776A double-mutant mice. Confirming our previous results, SCA1 S776A mice had an extension in life span of approximately 6–7 weeks (Figure 6F). This rescue was abolished in the S776A double-mutant mice (Figure 6F). Together, these data point toward a protective effect of the WT allele and raise the question about the mechanism behind the reduced rescue effect.

### Disruption of S776 phosphorylation on the WT allele reduces the transcriptional rescue seen in SCA1 S776A animals.

**Figure 7:**
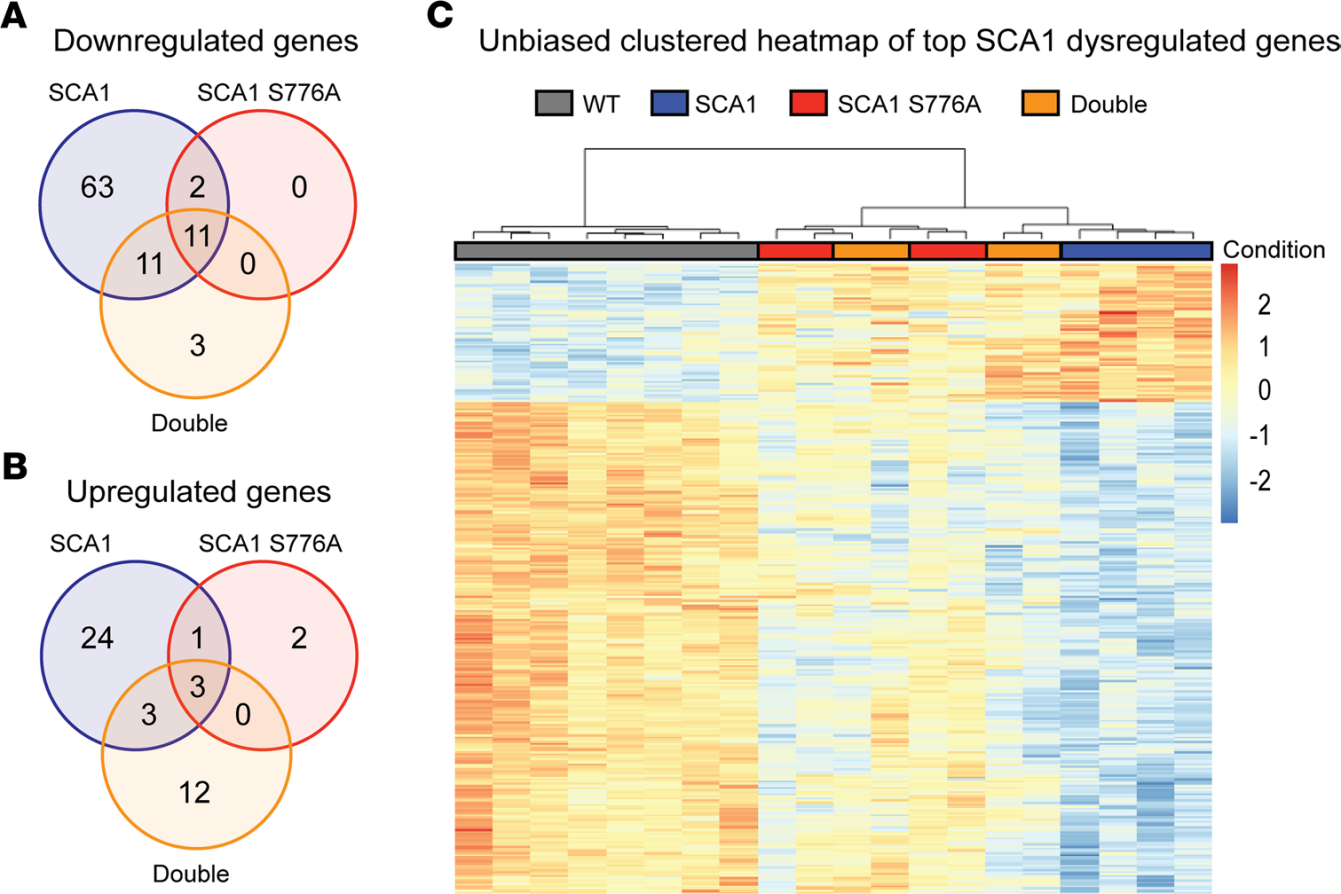
Transcriptional rescue is more pronounced in SCA1 S776A than in S776A double mutants. (**A**) Downregulated and (**B**) upregulated differentially expressed genes (DEGs) in the cerebellum of 6-week-old *Atxn1^154Q/2Q^* (SCA1) and *Atxn1^154Q[S776A]/2Q^* (SCA1 S776A) mice and *Atxn1^154Q[S776A]/2Q[S776A]^* (S776A Double) mice compared with *Atxn1^2Q/2Q^* (WT) mice (based on adjusted *P* value of < 0.01; absolute log_2_ fold change > 0.5). (**C**) Unbiased clustered heatmap of top SCA1 dysregulated genes (based on adjusted *P* value) of WT, SCA1, SCA1 S776A, and S776A double mutant mice. A total of 4–8 animals per genotype were used for the transcriptomic analysis.

To investigate the cause of the differential rescue effect of SCA1 S776A and S776A double mutants, we performed transcriptomic analysis on cerebella of WT, SCA1, SCA1 S776A, and S776A double mutants at 6 weeks of age. SCA1 mice had marked gene expression changes (87 downregulated and 31 upregulated) compared with WT, whereas SCA1 S776A mice showed drastically fewer transcriptional changes (13 downregulated and 6 upregulated; Figure 7, A and B). The S776A double mutants exhibited moderate gene expression changes, falling between SCA1 and SCA1 S776A (25 downregulated and 18 upregulated) and displayed additional expression changes that were not present in SCA1 or SCA1 S776A mice (Figure 7, A and B). Unbiased clustered heatmaps of the top dysregulated genes in SCA1 (based on adjusted *P* value) revealed separate clustering of WT, SCA1, and SCA1 S776A samples (Figure 7C). Importantly, S776A double-mutant samples were split and clustered with both SCA1 and SCA1 S776A samples (Figure 7C), further demonstrating a reduced transcriptional rescue of the S776A double mutants. These gene expression changes are consistent with our behavioral findings in that S776A double mutants display a reduced improvement.

The questions remained, however, as to why the transcriptional rescue was less pronounced in the S776A double mutants and why some new changes were observed. Two potential reasons could be a loss-of-function effect, owing to reduction of the WT allele or an effect attributed to the homozygous loss of S776 phosphorylation. To test if either reason could be causative for the reduced rescue, we repeated the grip strength, pole, and rotarod tests, as well as transcriptomic analysis on WT S776A homozygous mice, which have a complete loss of S776 phosphorylation and reduced ATXN1 levels (Supplemental Figure 6, A–C). We found that the WT S776A homozygous mice performed similar to WT in all behavioral tests (Supplemental Figure 6, A–C). Furthermore, the newly appearing transcriptional changes in the S776A double mutants did not overlap with the few expression changes seen in the WT S776A homozygous mice (Supplemental Figure 6D), and WT S776A homozygous samples clustered mainly with WT samples and not with S776A double mutants in the unbiased clustered heatmap of top SCA1 dysregulated genes (Supplemental Figure 6E).

These data suggest that homozygous loss of S776 phosphorylation and the resulting decrease of WT ATXN1 do not drastically affect behavior and gene expression in mice. Instead, reduction of the WT allele appears to only alter behavior and gene expression in the presence of the polyQ-expanded allele, indicating that WT ATXN1 confers protective effects that are specific to the context of SCA1.

## Discussion

In this study, we evaluated the potential of targeting ATXN1 S776 phosphorylation as a therapeutic entry point for SCA1. We found that ATXN1 is phosphorylated at S776 across various brain regions and peripheral tissues and showed that the phosphorylation event is critical for ATXN1 stability in the cerebellum and throughout all brain regions affected in SCA1. As previous studies have shown the benefits of disrupting S776 phosphorylation and reducing ATXN1 levels in the cerebellum (9), we investigated the effects of abolishing S776 phosphorylation on all behavioral phenotypes seen in SCA1. Disruption of S776 phosphorylation exclusively on the polyQ-expanded ATXN1 rescued not only the SCA1 motor coordination deficits but also improved the respiratory dysfunction and extended the survival of SCA1 mice. Interestingly, whereas polyQ-expanded ATXN1 was reduced in the hippocampus, we did not detect an improvement of the SCA1 learning and memory phenotypes. Furthermore, the weight loss and kyphosis phenotypes were not improved. As the cerebellum shows the greatest benefit from preventing S776 phosphorylation and as not all SCA1 phenotypes are improved, this might also suggest that distinct disease mechanisms contribute to ATXN1 toxicity in different affected tissues.

Although we detected a therapeutic benefit of disrupting S776 phosphorylation on polyQ-expanded ATXN1, therapeutic interventions, such as kinase inhibitors, would likely not only lower polyQ-expanded ATXN1 but also affect WT ATXN1 levels. Therefore, we compared the rescue effect of disrupting S776 phosphorylation and reducing polyQ-expanded ATXN1 alone or in combination with WT ATXN1. We found that disruption of S776 phosphorylation on both alleles reduced the rescue of the motor coordination phenotypes and abolished the survival benefit. Furthermore, whereas disruption of S776 phosphorylation on polyQ-expanded ATXN1 alone strongly improved the transcriptional changes observed in SCA1, we also observed a reduced transcriptional rescue when phosphorylation was disrupted on both alleles. Together, these data indicate that reducing S776 phosphorylation on both alleles is safe but reduces the rescue effect, thereby suggesting a neuroprotective role of WT ATXN1.

**Figure 8:**
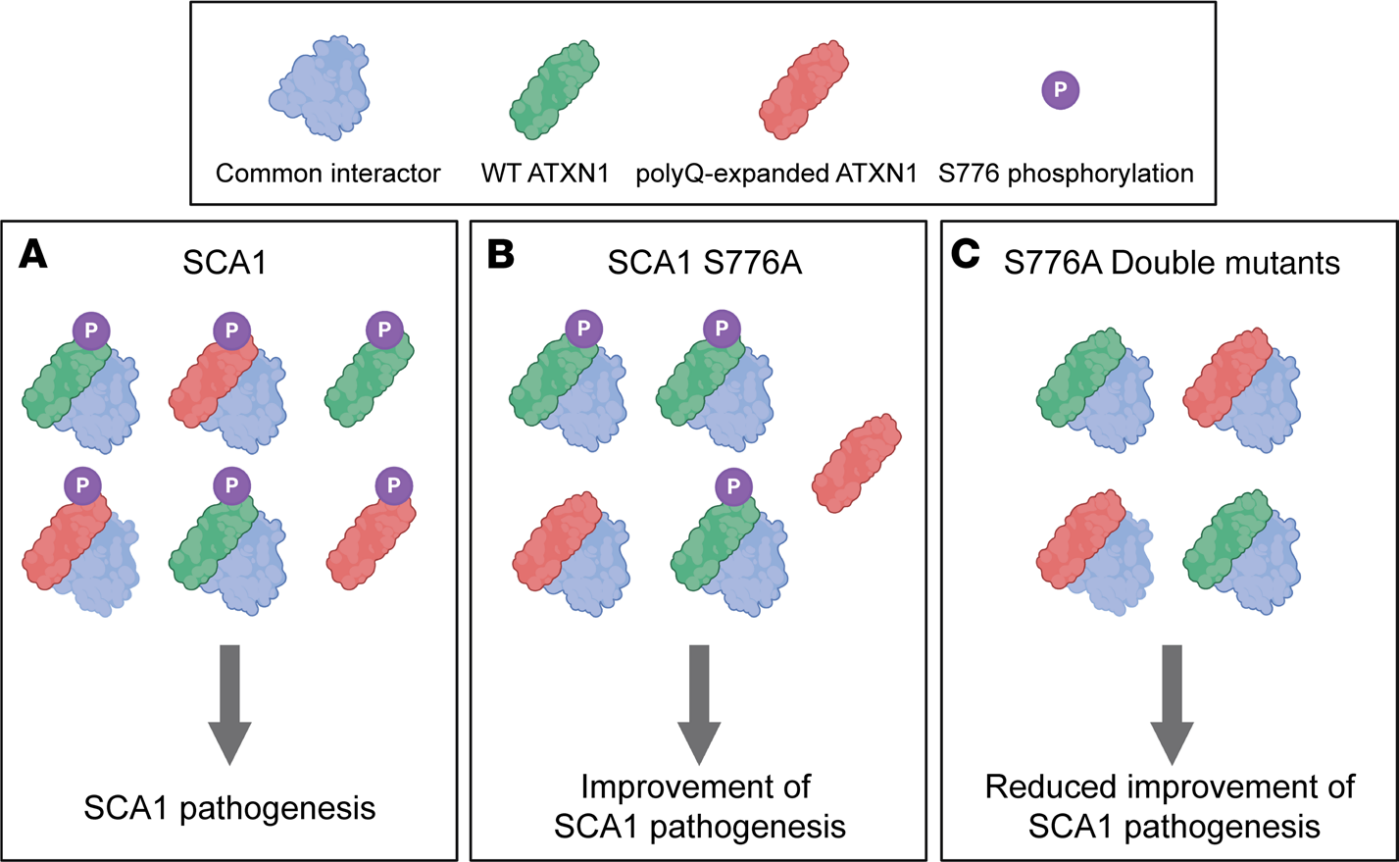
Proposed model for the neuroprotective function of WT ATXN1 in SCA1. The model illustrates the association of polyQ-expanded ATXN1 and WT ATXN1 into common complexes in *Atxn1^154Q/2Q^* (SCA1) and *Atxn1^154Q[S776A]/2Q^* (SCA1 S776A) and *Atxn1^154Q[S776A]/2Q[S776A]^* (S776A double mutants). (**A**) SCA1: WT ATXN1 competes with polyQ-expanded ATXN1 for the association with common interactors and forms toxic complexes leading to SCA1 pathogenesis. (**B**) SCA1 S776A: Disruption of S776 phosphorylation on the polyQ-expanded ATXN1 decreases its levels and reduces the formation of toxic complexes, resulting in an improvement of SCA1 pathogenesis. (**C**) S776A double mutants: Disruption of S776 phosphorylation on both alleles decreases polyQ-expanded ATXN1 and WT ATXN1. The reduction of WT ATXN1 decreases its association with common interactors, which then interact with polyQ-expanded ATXN1 to form toxic complexes, thereby reducing the rescue effect. The illustration was created using BioRender.com.

Interestingly, previous work showed that *ATXN1^–/–^* mice do not develop ataxia and have a normal life span (23), indicating that the reduced rescue effect is not attributed to a loss of function of ATXN1. Furthermore, total disruption of S776 phosphorylation in WT mice did not result in behavioral or transcriptional abnormalities, demonstrating that S776 phosphorylated WT ATXN1 is only protective in the context of SCA1. Similarly, previous reports showed a worsening of SCA1 motor coordination and survival phenotypes in *ATXN1^154Q/–^* mice (18). Because the increase of ATXN1 paralog, Ataxin-1-like, suppresses SCA1 neuropathology and improves SCA1 phenotypes by displacing polyQ-expanded ATXN1 from its native complexes (24), a potential mechanism explaining the neuroprotective effects of WT ATXN1 could be a shift in the balance of ATXN1 complexes. Lowering WT ATXN1 might free common interactors, resulting in an increase of toxic polyQ-expanded ATXN1–containing complexes (Figure 8). Together, these data point toward a neuroprotective effect of WT ATXN1, specifically in the context of SCA1, and call for the development of allele-specific strategies to reduce polyQ-expanded ATXN1.

To our knowledge, the present study is the first to report a differential rescue effect when comparing allele-specific and -nonspecific methods to reduce protein levels in a polyglutamine expansion disorder. Future studies will be required to explore the exact mechanism by which WT ATXN1 yields neuroprotection in SCA1 and to investigate if the neuroprotective function of the WT allele can also be found in other expansion disorders.

## Methods

### Mouse husbandry

*Atxn1^154Q/2Q^* (SCA1) mice (19) and *Atxn1*^–*/*–^ (*Atxn1*-KO) mice (23) were backcrossed to C57BL/6J background for a minimum of 10 generations. All mice were housed and maintained in the animal facilities at Baylor College of Medicine. The mice were kept on a 12-hour light/12-hour dark cycle.

### Generation of *Atxn12Q[S776A]/2Q* and *Atxn1154Q[S776A]/2Q* mouse models

*Atxn1^2Q[S776A]/2Q^* and *Atxn1^154Q[S776A]/2Q^* mice were generated via CRISPR/Cas9-mediated gene editing (25, 26). The gRNA (5′-GGCGCCGGAGACCCGTAAAC-3′) was selected based on the highest quality score and a low number of off-target sites using the http://crispr.mit.edu page. To generate a sgRNA targeting exon 8 of mouse *Atxn1*, we first synthesized the DNA template by direct PCR from the pX459 v2.0 vector (Addgene 62988) using the Phusion High-Fidelity DNA Polymerase (New England Biolabs) and the primers gRNA_S776A_For (5′-TTAATACGACTCACTATAGGGGCGCCGGAGACCCGTAAACGTTTTAGAGCTAGAAATAGC-3′) and gRNA_S776A_Rev (5′-AAAAGCACCGACTCGGTGCC-3′). The reaction was run on a low melting 2% MetaPhor Agarose gel (Lonza) at 80 V at 4°C. The corresponding band was excised and purified using the Wizard SV Gel and PCR Clean-Up System (Promega). The PCR product was then in vitro transcribed using the MEGAshortscript T7 Transcription kit (Invitrogen), and the gRNA was purified using the MEGAclear Transcription Clean-Up Kit (Thermo Fisher Scientific).

A single-stranded oligonucleotide (ssODN) was purchased from Integrated DNA Technologies for homologous-directed recombination to introduce the S776A and additional synonymous mutations in *Atxn1* (5′-ACGTTAGATCGGCCTTCGATGCAGATCTTAACCTCCTGAGGAATGAGCGAAGGCTTGGGAAGAGTCAAAGGTGGCTCGTCCTCCGAtTTtTCgAGcTTtCGtGTtTCgGGGCCGcCCAtCTCCTCTTCCTCGTGGCTGTGGGTTTGCTGG-3′; modified nucleotides are written in lowercase). The additional synonymous mutations were used to alter the protospacer adjacent motif to increase editing efficiency as well as to allow for simple genotyping by differential primer hybridization.

Female C57BL/6J WT mice (3–4 weeks of age) were purchased from The Jackson Laboratory and super-ovulated via injection of 5 IU PMSG (Thermo Fisher Scientific) and 48 hours later with 5 IU HCG (Thermo Fisher Scientific) in 0.9% NaCl. Upon overnight breeding with *Atxn1^154Q/2Q^* males, the females’ ova were dissected. An injection mixture containing 30 ng/μL Cas9 protein (PNA Bio), 20 ng/μL sgRNA, and 40 ng/μL ssODN in 10 mM Tris, pH 7.5, and 0.25 mM EDTA was prepared, and 2 pL was injected into the pronucleus of extracted ova. The ova were then transferred into oviducts of pseudopregnant FVB females by the Baylor College of Medicine Genetically Engineered Mouse Core.

Upon birth, the following primers were used to distinguish unmodified (WT) and modified (S776A) alleles: WT/S776A_CRISPR_For (5′-CCAAACTCTCTGTTGGGGACGTCTGCATCTCGCTCAC-3′), WT_CRISPR_Rev (5′-TTCTCCAGTTTACGGGTCTCCGGCGCCGA-3′) and S776A_CRISPR_Rev (5′-GATTTTTCGAGCTTTCGTGTTTCGGGTGCCGCCCATCTC-3′). Note that the forward primer is the same for both reactions. Positive founders were backcrossed to C57BL/6J WT animals (The Jackson Laboratory), and the correct sequence of the F1 offspring was confirmed using Sanger sequencing (Genewiz). The mice were backcrossed for a minimum of 3 generations before conducting molecular and behavioral assays to prevent off-target effects.

### RNA extraction and RT-qPCR

RNA from different brain regions was isolated from 6-week-old mice (*n* = 6 per genotype). Total RNA was isolated using the miRNeasy Mini Kit from Qiagen following the manufacturer’s instructions. RNA was quantified using NanoDrop 1000 (Thermo Fisher Scientific), and random-primed cDNA was prepared from 1 μg total RNA using M-MLV Reverse Transcriptase (Invitrogen). RT-qPCR was then performed with PowerUp SYBR Green Master Mix (Applied Biosystems), and samples were run on a real-time PCR detection system (Bio-Rad CFX96). All samples were analyzed in triplicate, and *Atxn1* expression levels were normalized to the expression of the housekeeping gene *Gapdh*. Primers for *Gapdh* and *Atxn1* were obtained from MilliporeSigma. Primers used for *Gapdh* were Gapdh_For (5′- AGGTCGGTGTGAACGGATTTG-3′) and Gapdh_Rev (5′-TGTAGACCATGTAGTTGAGGTCA-3′) and primers used for *Atxn1* were Atxn1_For (5′-GAGAATCGAGGAGAGCCAC-3′) and Atxn1_Rev (5′- AGACTTCGACACTGACCTG -3′).

### Protein extraction and Western blot of brain tissues

Homogenates of different brain regions from 4- to 6-week-old mice (*n* = 6 per genotype) were prepared by Dounce homogenization in NETN buffer (100 mM NaCl, 20 mM Tris-HCl, pH 8.0, 0.5 mM EDTA, 1.5% NP-40, 1× protease inhibitor from Roche, 1× phosphatase inhibitor from MilliporeSigma). Samples were sonicated for a total of 10 times, incubated for 30 minutes at 4°C, and spun at 13,000 rpm at 4°C for 15 minutes. Protein concentrations of the supernatant were measured using the Pierce BCA Protein Assay Kit (Thermo Fisher Scientific). Samples were diluted and prepared in NuPAGE sample reducing agent (Invitrogen) and NuPAGE LDS Sample Buffer (Invitrogen). The samples were then boiled for 10 minutes and then run on NuPAGE 4%–12% Bis-Tris gel 1.5 mm 15-well gels (Invitrogen) in MES running buffer (50 mM MES, 50 mM Tris base, 0.1 % SDS, 1 mM EDTA). The proteins were subsequently transferred to Immobilon-FL PVDF membranes (Invitrogen, 0.45 μm). After blocking for 1 hour at room temperature with 1:1 Odyssey Blocking Buffer TBS (LI-COR Biosciences) in TBS, membranes were probed overnight at 4°C with anti-ATXN1 (11750VII, 1:2000; ref. 27), anti-ATXN1-S776 (PN1168; 1:1000; ref. 9), and anti-GAPDH (MilliporeSigma; 6C5, 1:10,000) in 1:1 Odyssey Blocking Buffer and TBS (LI-COR Biosciences) in Tris-buffered saline (5 mM Tris, pH 7.5, 120 mM NaCl) with 0.1% Tween-20 (TBST). The secondary antibody used to detect anti-ATXN1 and anti-ATXN1-S776 was Rabbit IgG (H&L) Antibody DyLight 680 Conjugated (Rockland Immunochemicals; 611-144-002; 1:10,000), and Mouse IgG (H&L) Antibody DyLight 800 Conjugated (Rockland Immunochemicals; 610-145-002; 1:10,000) was used to detect anti-GAPDH in 1:1 Odyssey Blocking Buffer and TBS (LI-COR Biosciences) in TBST. The membranes were washed 3 times with TBST and then imaged using the Odyssey CLx Imaging System (LI-COR Biosciences).

### Histology

For cerebellar sections, mouse brains were collected at 44 weeks of age and fixed for 24 hours in 10% formalin. The brains were transferred to 15% sucrose in PBS (137 mM NaCl, 2.7 mM KCl, 10 mM NA_2_HPO_4_, 1.8 mM KH_2_PO_4_) for 24 hours and then placed in 30% sucrose in PBS. Once the brains had sunk, they were embedded in OCT media. Cerebellar sagittal sections were cut to a thickness of 40 μm. Free-floating sections were washed 3 times in PBS and blocked in blocking solution (10% goat serum in PBS with 0.2% Tween-20) for 2 hours at room temperature. Cerebellar sections were immunostained with primary anti-calbindin D-28k antibody (Swant; 300; 1:1000) in blocking solution overnight at 4°C. Upon 3 washes with PBS, the sections were incubated with Alexa Fluor 488 Goat anti-mouse IgG (H+L) (Invitrogen; A11001; 1:1000) for 2 hours at room temperature. The sections were washed 3 times with PBS and then stained with DAPI (1:2000; Thermo Fisher Scientific) in PBS for 5 minutes at room temperature. The sections were washed again 3 times with PBS, mounted using ProLong Gold antifade reagent (Invitrogen), and imaged using the Zeiss LSM 710 confocal microscope. Quantification of molecular layer thickness was performed using ImageJ software (NIH).

For the staining of astrocytes in the hypoglossal nucleus, brain tissue was collected from 30-week-old animals and drop fixed in 10% formalin for 24 hours. Standard techniques were used to embed tissue in paraffin and to take coronal sections 10 μm thick in serial sections every 100 μm. Sections were then prepared for staining by being deparaffinized and rehydrated. Slides were treated with antigen retrieval by boiling for 9 minutes in 10 mM sodium citrate, 0.05% Tween 20, pH 6.0. GFAP antibody (MilliporeSigma; G3893; 1:500) was incubated for approximately 12 hours at 4°C. This primary antibody was then detected with biotinylated horse anti-mouse IgG (Vector Laboratories; 1:1000) and visualized using an ABC reagent kit (Vector Laboratories) according to the manufacturer’s recommendations. Brightfield images were acquired using a Carl Zeiss Axio Imager M2 microscope, equipped with an Axio Cam MRc5 color camera (Carl Zeiss). All immunohistochemistry experiments were performed in triplicate, and figures show representative results. Astrocyte density was determined by counting the number of astrocytes per region of interest (defined as an area of 90,000 μm^2^).

### RNA sequencing

Cerebella tissue from 6-week-old mice (*n* = 4–8 per genotype) were dissected. Total RNA was isolated using the miRNeasy Mini Kit (Qiagen) following manufacturer’s instructions. RNA was submitted to Genewiz for RNA integrity assessment, library preparation, and sequencing on the Illumina HiSeq platform. For each sample, approximately 100 million pairs of 150 bp pair-end reads were generated. Raw reads were trimmed before mapping by Trimmomatic-0.36 using the adapter reference TruSeq3-PE.fa (28). STAR v2.7.2d was used to align trimmed reads to the *Mus musculus* genome (GRCm38.p6, https://www.ncbi.nlm.nih.gov/assembly/GCF_000001635.26) and to obtain read counts with default parameters (29). The mappabilities for all 24 samples were above 89.8%.

Next, as the 24 samples were sequenced in 2 batches, the read counts were batch corrected using the ComBat_seq function in the SVA Package v3.36.0 (30), including biological covariates for genotype. The corrected reads were then used for differential gene expression analysis using the DESeq2 package v1.28.1 (31). Heatmaps were generated using the 300 genes with lowest adjusted *P* values using the pheatmap package v1.0.12 (32). Specifically, the heatmaps show unbiased clustering plotting *z* scores for each gene, by normalizing the row to have an average expression of 0 and an SD of 1. The RNA sequencing data can be accessed at the National Center for Biotechnology Information’s Gene Expression Omnibus database under accession number GSE163885.

### Behavioral tests

All behavioral analyses were performed during the light phase of the 12-hour light/12-hour dark cycle by an individual who was blinded to the genotypes of the mice. The mice had access to food and water ad libitum except during tests. All mice were age matched within experiments, and littermate controls were used when possible. For each test, the mice were habituated for 30 minutes in the test room before testing. Testing was done at a room brightness of 200 lux with white noise playing at 60 dB.

#### Grip strength test.

The grip strength test was performed at 7 weeks of age. The mice were held by the tail and allowed to grab the bar of the grip strength meter (Columbus Instruments) with both forepaws. The mice were pulled away from the bar with a constant slow force until the forepaws released. The maximum force generated was averaged over 3 pulls for each mouse.

#### Pole test.

The pole test was performed at 7 weeks of age. Each mouse was placed head-upward at the top of a 60.5 cm tall vertical threaded metal pole. Time of descent was measured with a 60-second cutoff time. Falls from the metal pole were counted as a 60-second descent. In case the mouse fell off the pole within the first 10 seconds, the trial was not counted, and the mouse was placed on the pole again to ensure proper grip. The test was performed in triplicate for each animal.

#### Rotarod test.

The rotarod test was performed at 7 and 24 weeks of age to evaluate coordination and motor skill acquisition (type 7650; Ugo Basile). The mice were placed on the rotating rod (3 cm diameter, 30 cm long) for 4 trials every day for a period of 4 days. Each trial lasted a maximum of 10 minutes. The rod accelerated from 4 to 40 rpm in 5 minutes and remained at 40 rpm for the remaining 5 minutes. The time the mice took to fall was recorded. Two subsequent rotations around the rod were also counted as a fall.

#### Plethysmography.

Whole-body plethysmography (Buxco) was performed as described previously (6). Mice at 24 and 42 weeks of age were habituated to the chambers for 1 hour before recording the respiration for 30 minutes. Air was pumped through the chambers at a rate of 0.5 L/min. Breath waveforms were identified using Phonemah 3 software (DSI), and the breathing rate and tidal volume were subsequently analyzed using a customized MATLAB (MathWorks) code. As mouse movements can induce artifacts, breaths with an inspiratory time under 0.025 seconds, expiratory time over 10 seconds, or calculated expiratory tidal volume more than twice the inspiratory tidal volume were excluded. A sliding window of 200 breaths was used to filter out intervals during which more than 10% of breaths were taken at a rate faster than 600 breaths per minute. Interbreath interval irregularity (IBII) was defined as IBII = abs [breath length (*n*+1) − breath length (*n*)]/breath length (*n*), where abs is equal to the absolute value and *n* is equal to the chronological number of a recorded breath. Mice with fewer than 100 reliable recorded breaths were excluded from the analysis.

### Statistics

Experimental analysis was performed in a blinded manner when possible. Statistical tests were performed in accordance with the experimental design. Simple comparisons used Student’s 2-tailed *t* test, whereas multigroup comparisons used 1- or 2-way ANOVAs. Survival analysis used log-rank test. In each case, *P* < 0.05 was considered statistically significant.

### Study approval

Animal care and experimental procedures were approved by the IACUC of Baylor College of Medicine, according to NIH guidelines.

## Author contributions

LN, HTO, and HYZ conceived the study, designed experiments, and analyzed and interpreted data. LN performed molecular and behavioral assays and wrote the manuscript. LN and HYZ edited the manuscript. SLC, EX, and DBE performed molecular and behavioral experiments. JPO performed histology assays. EA assisted with molecular assays. SLC, YD, YWW, and ZL further performed RNA sequencing analysis. All authors reviewed the manuscript and provided input.

## Supplementary Material

Supplemental data

## Version 1 02/08/2021

Electronic publication
