# Supplementary material for: Modulation of ATXN1 S776 phosphorylation reveals the importance of allele-specific targeting in SCA1
Source: JCI Insight. 2021 Feb 8;6(3):e144955. doi: 10.1172/jci.insight.144955 (PMC7934855; doi:10.1172/jci.insight.144955)
Supplement: Supplemental data [file jciinsight-6-144955-s047.pdf]

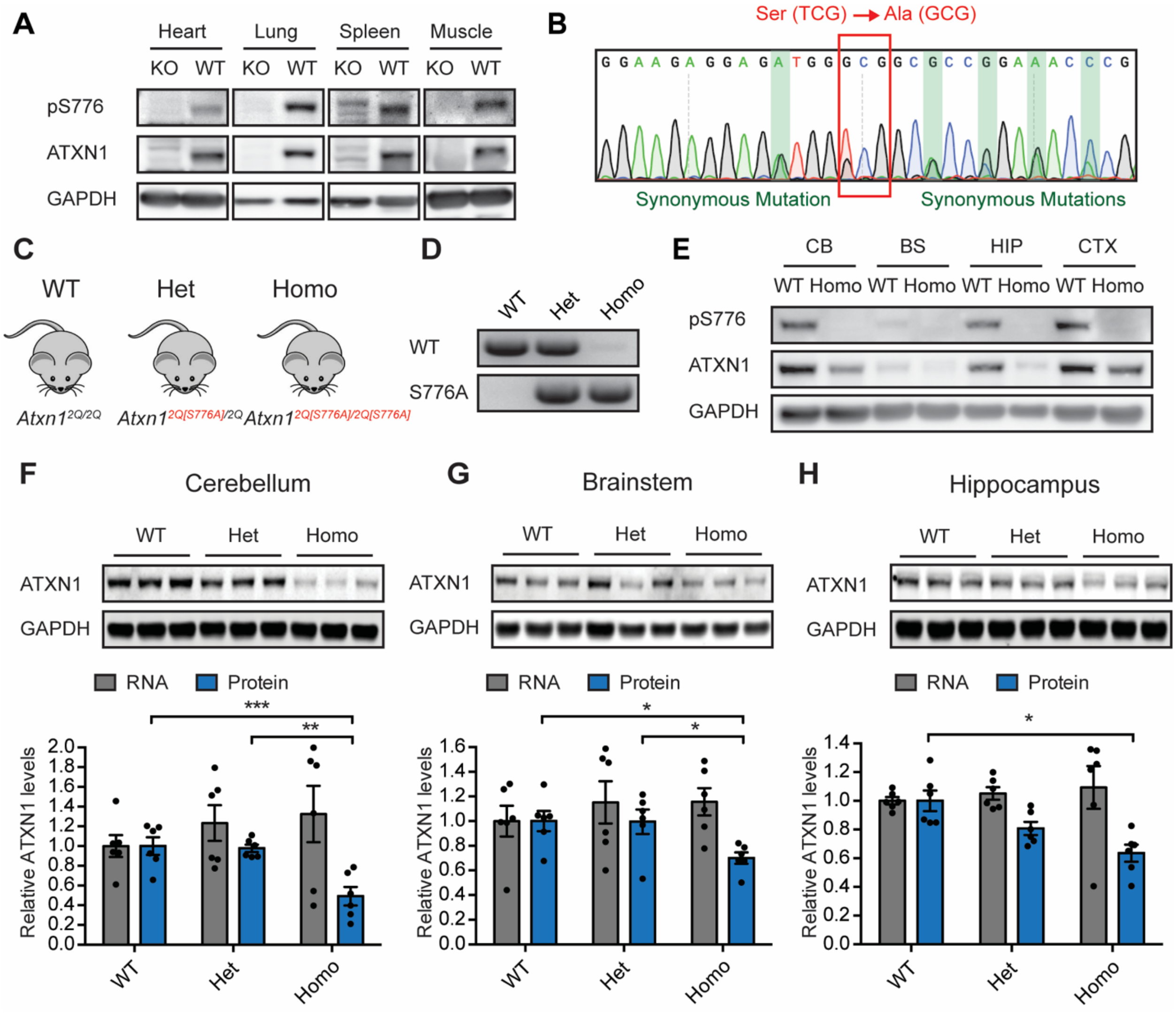

Supplemental Figure 1. Disruption of S776 phosphorylation reduces ATXN1[2Q] levels in the cerebellum, brainstem, and hippocampus in offspring of the second founder. A) Representative western blot showing S776 phosphorylation in the heart, lung, spleen, and muscle of *Atxn1*$^{-/-}$ (KO) and *Atxn1*$^{2Q/2Q}$ (WT) mice. **B)** Sanger sequencing confirming S776A and synonymous mutations in heterozygous F1 offspring of the second founder following CRISPR injections. **C)** Genotypes of experimental animals: *Atxn1*$^{2Q/2Q}$ (WT), *Atxn1*$^{2Q[S776A]/2Q}$ (Het) and *Atxn1*$^{2Q[S776A]/2Q[S776A]}$ (Homo). **D)** Genotyping for WT, Het and Homo mice using specific primers for WT and S776A allele. **E)** Representative western blot confirming loss of S776 phosphorylation in Homo mice from second founder. Representative western blots and quantification of *Atxn1* RNA and protein levels in **F)** the cerebellum, **G)** brainstem and **H)** hippocampus of 6-week old WT, Het and Homo mice from the second founder. For each assay, a minimum of 3 replicates

were performed. Multi-group comparisons used one-way ANOVAs. In each case, *, **, ***, **** and ns denote $p<0.05$, $p<0.01$, $p<0.001$, $p<0.0001$ and $p>0.05$, respectively. All data are represented as means ± SEM.

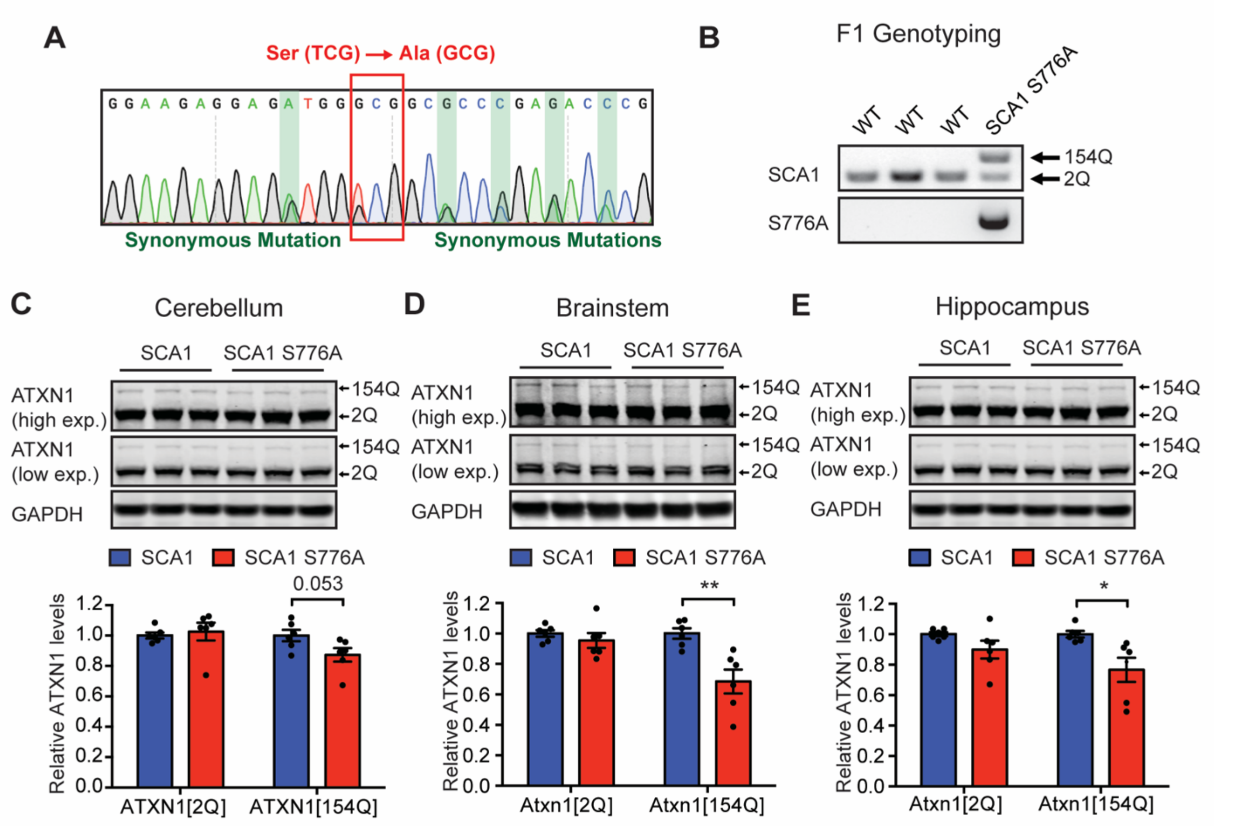

Supplemental Figure 2. Disruption of S776 phosphorylation reduces polyQ-expanded ATXN1 levels in the cerebellum, brainstem and hippocampus in offspring of the second founder. A) Sanger sequencing confirming S776A and synonymous mutations in heterozygous F1 offspring of the second SCA1 S776A founder. **B)** Genotyping of F1 offspring using specific primers to distinguish WT and SCA1 animals as well as primers for the detection of the S776A allele. Representative western blots and quantification of ATXN1[2Q] and ATXN1[154Q] protein levels in **C)** the cerebellum, **D)** brainstem and **E)** hippocampus of 6-week old *Atxn1*$^{154Q/2Q}$ (SCA1) and *Atxn1*$^{154Q[S776A]/2Q}$ (SCA1 S776A) mice. For each assay, a minimum of 6 replicates were performed. Simple comparisons used Student's t-test. In each case, *, **, ***, **** and ns denote $p<0.05$, $p<0.01$, $p<0.001$, $p<0.0001$ and $p>0.05$, respectively. All data are represented as means ± SEM.

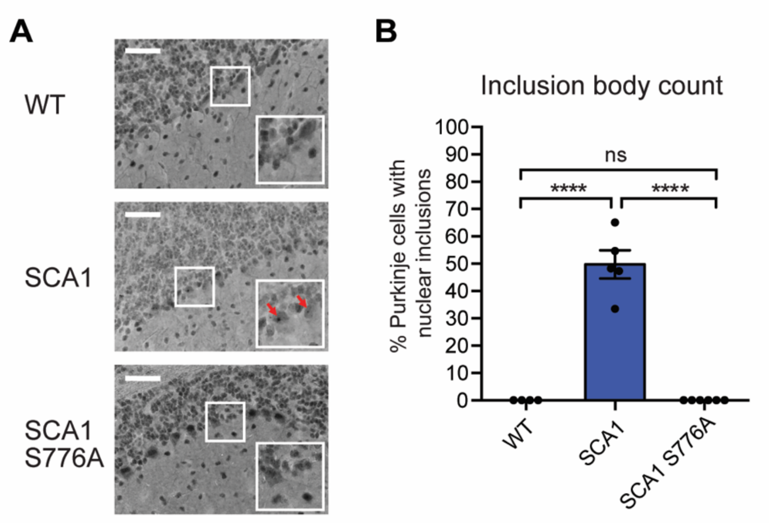

Supplemental Figure 3. Disruption of S776 phosphorylation abolishes ATXN1 nuclear inclusion body formation. A) Immunofluorescent staining of the cerebellar cortex with anti-ATXN1 antibody 11NQ in *Atxn1*$^{2Q/2Q}$ (WT), *Atxn1*$^{154Q/2Q}$ (SCA1) and *Atxn1*$^{154Q[S776A]/2Q}$ (SCA1 S776A) mice at 37-weeks of age. Scale bar represents 40μm. **B)** Quantification of the percentage of Purkinje cells with nuclear inclusions in 4-6 mice per genotype. Multi-group comparison used one-way ANOVAs. In each case, *, **, ***, **** and ns denote $p<0.05$, $p<0.01$, $p<0.001$, $p<0.0001$ and $p>0.05$, respectively. All data are represented as means ± SEM.

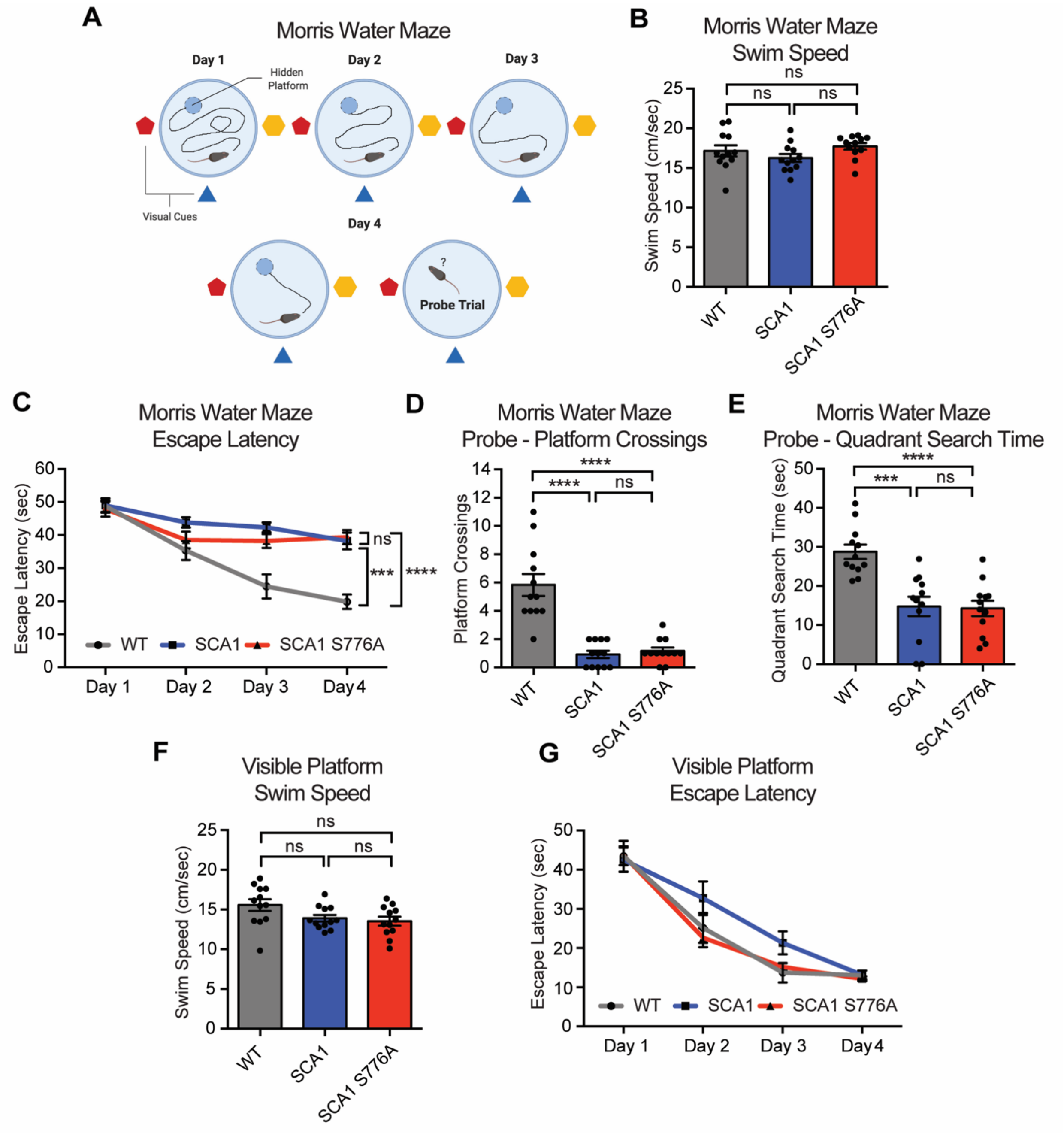

Supplemental Figure 4. Disruption of S776 phosphorylation on polyQ-expanded ATXN1 has no effect on SCA1 learning and memory deficits. A) Schematic overview of the Morris Water Maze Test. **B)** Swim Speed on day 1 of the Morris Water Maze of *Atxn1*$^{2Q/2Q}$ (WT), *Atxn1*$^{154Q/2Q}$ (SCA1) and *Atxn1*$^{154Q[S776A]/2Q}$ (SCA1 S776A) mice at 14 weeks of age. **C)** Escape latency in the Morris Water Maze. **D)** Platform crossings and **E)** quadrant search time in the probe trail of the Morris Water Maze. **F)** Swim speed during the first day of the Visible Platform test. **G)** Escape latency in the Visible Platform test. For each assay a minimum of 12 animals per genotype were

used in each test. Multi-group comparisons used one- and two-way ANOVAs. In each case, *, **, ***, **** and ns denote $p<0.05$, $p<0.01$, $p<0.001$, $p<0.0001$ and $p>0.05$, respectively. All data are represented as means ± SEM. The illustration was created using Biorender.com.

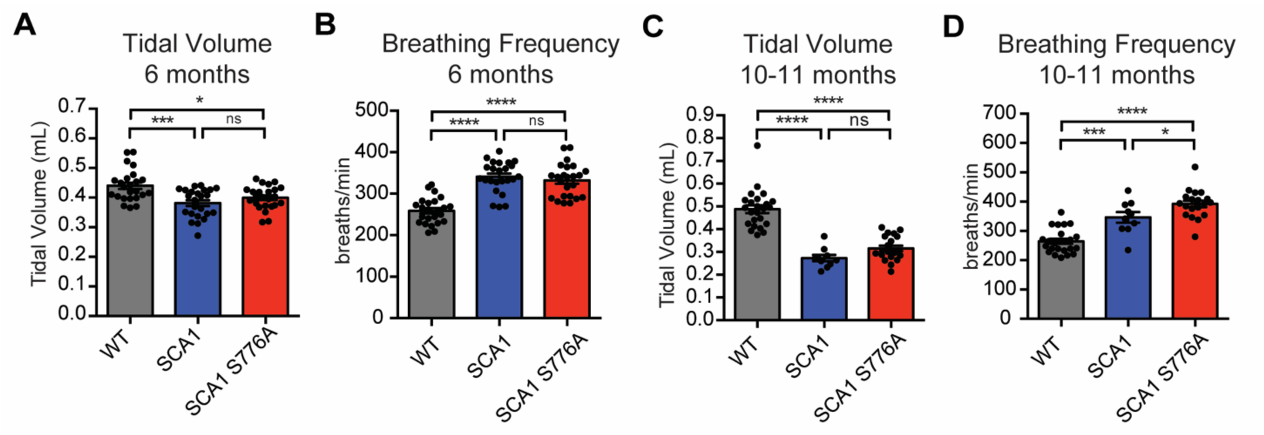

Supplemental Figure 5. Disruption of S776 phosphorylation on polyQ-expanded ATXN1 has no effect on SCA1 breathing dysfunction at 6 months of age. A, C) Tidal volume and **B, D)** breathing frequency of $Atxn1^{2Q/2Q}$ (WT), $Atxn1^{154Q/2Q}$ (SCA1) and $Atxn1^{154Q[S776A]/2Q}$ (SCA1 S776A) mice at **A, B)** 6 months and **C, D)** 10-11 months of age. A minimum of 10 animals per genotype was used. Multi-group comparisons used one-way ANOVAs. In each case, *, **, ***, **** and ns denote $p<0.05$, $p<0.01$, $p<0.001$, $p<0.0001$ and $p>0.05$, respectively. All data are represented as means ± SEM

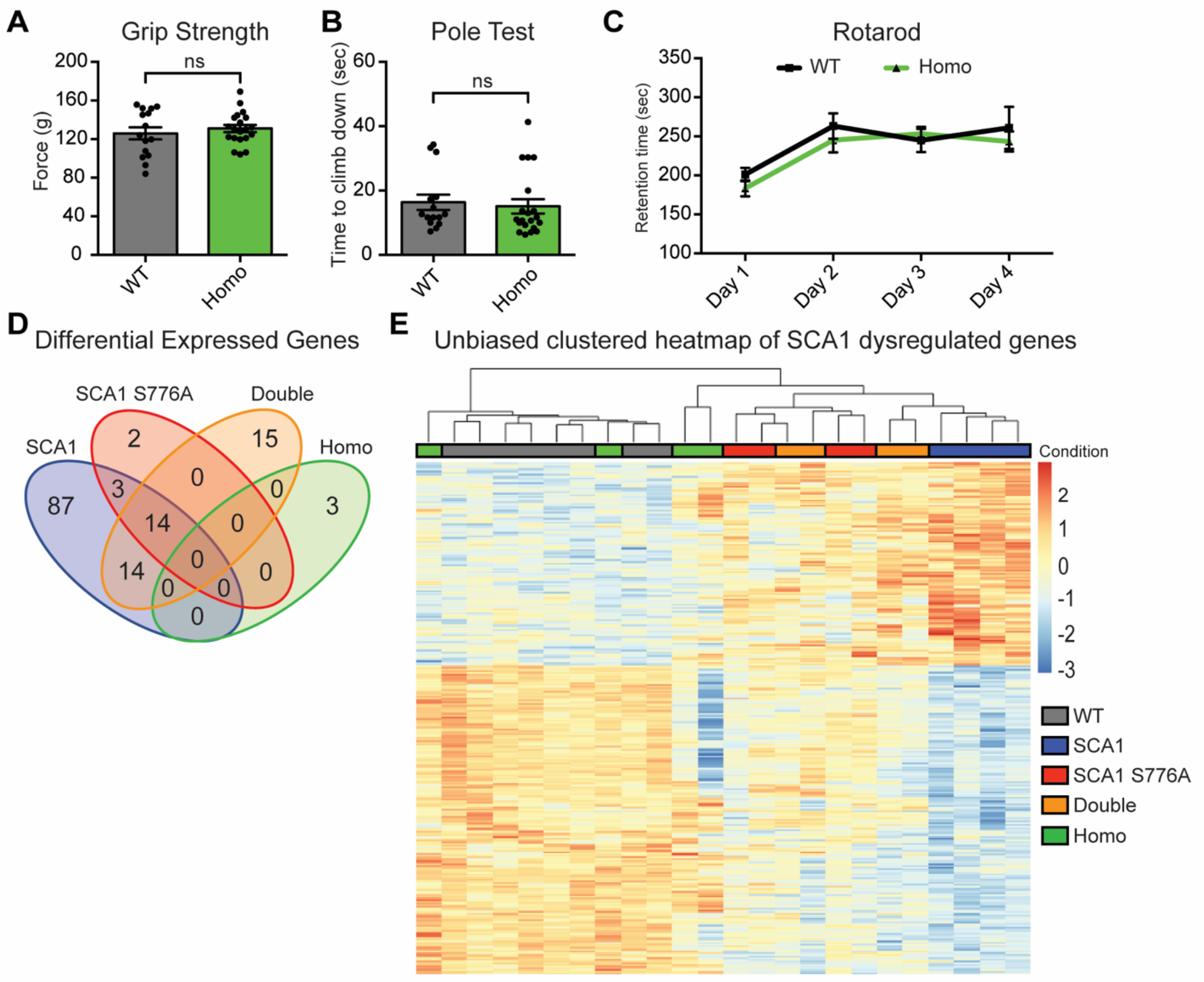

Supplemental Figure 6. WT S776A homozygous mice do not display SCA1-specific behavioral and transcriptional changes. A) Grip strength, **B)** Pole test and **C)** Rotarod of *Atxn1*$^{2Q/2Q}$ (WT) and *Atxn1*$^{2Q[S776A]/2Q[S776A]}$ (Homo) mice at 7 weeks of age. A minimum of 15 animals per genotype were used for behavioral assays. **D)** Differentially expressed genes (DEGs) in the cerebellum of 6-week old *Atxn1*$^{154Q/2Q}$ (SCA1), *Atxn1*$^{154Q[S776A]/2Q}$ (SCA1 S776A) mice, *Atxn1*$^{154Q[S776A]/2Q[S776A]}$ (S776A Double) and *Atxn1*$^{2Q[S776A]/2Q[S776A]}$ (Homo) mice (based on p-adjusted value of <0.01; absolute(log2 fold change) >0.5). **E)** Unbiased clustered heatmap of top SCA1 dysregulated genes (based on p-adjusted value) of 6-week old WT, SCA1, SCA1 S776A, S776A Double mutant and Homo mice. 4-8 animals per genotype were used for the transcriptomic analysis. Simple comparisons used Student's t-test and multi-group comparisons used two-way ANOVAs. In each case, *, **, ***, **** and ns denote p<0.05, p<0.01, p<0.001, p<0.0001 and p>0.05, respectively. All data are represented as means ± SEM.

Supplemental Materials and Methods

Protein extraction and western blot of peripheral tissues

For peripheral tissues, the tissues were homogenized in NETN buffer ((100mM NaCl, 20mM Tris-HCl, pH 8.0, 0.5mM EDTA, 1.5% NP-40, 1X protease inhibitor (Roche), 1X phosphatase inhibitor (Sigma)) using the Tissue Lyser II (Qiagen). Samples were incubated for 30 min at 4°C, sonicated 10 times, and briefly centrifuged at 13,000 rpm at 4°C for 15 min. Protein concentrations of the supernatant were measured using the Pierce BCA Protein Assay Kit (Thermo Fisher). Supernatants were then prepared with NuPAGE sample reducing agent (Invitrogen) and NuPAGE LDS Sample Buffer (Invitrogen). The samples were boiled for 10 min and then run on NuPAGE 4-12% Bis-Tris Gel 1.5mm 15-well gels (Invitrogen) in MES Running Buffer (50 mM MES, 50 mM Tris base, 0.1 % SDS, 1 mM EDTA). The proteins were subsequently transferred to Amersham Protran 0.2 NC nitrocellulose western blotting membranes (GE Healthcare Life Sciences) using Tris-Glycine Transfer Buffer (25 mM Tris Base, 192 mM Glycine, 10% Methanol). The membranes were then blocked at room temperature for 1 hr using 3% BSA in TBST. The membranes were probed overnight at 4°C with anti-ATXN1 (11750VII, 1:2,000) or anti-GAPDH (Sigma, 6C5, 1:10,000) in 3% BSA in TBST. The next day, the membranes were washed three times with TBST and then incubated for 1 hr at room temperature with either Immun-Star Goat anti-Rabbit HRP Conjugate (BioRad; 170-5046; 1:10000) to detect ATXN1 or AffiniPure anti-Mouse HRP Conjugate (Jackson ImmunoResearch Laboratories; 715-035-150; 1:10000) to detect GAPDH. The membranes were then washed three times with TBST, incubated for one minute in Amersham ECL Prime reagent (Thermo Fisher), and then imaged using ImageQuant LAS 4000 Imager (GE Healthcare Life Sciences).

Behavioral tests

Morris Water Maze and Visual Platform Test

At 14 weeks-of-age the mice were trained in the Morris Water Maze task to locate a hidden platform in a circular pool (140 cm diameter) of opaque water. Each mouse was trained with two blocks of four trials per day spaced 30 min apart for four consecutive days. In each training trail, the mice were released from a different starting point and could search for the hidden platform (10 cm in diameter) for 60 s. If a mouse did not find the platform within 60 s, the mouse was guided to the platform and remained on the platform for 10 s before being removed from the pool. On the fourth and final training day, memory was assessed in a probe trial during which the platform was removed, and the mice were allowed to search for 60 s. Data were acquired and analyzed using the EthoVision System (Nodulus).

The visible platform test was performed on a separate cohort of 14-week old mice using the same procedures as described for the Morris Water Maze, with the exception that a black cube was attached to the top of the escape platform. The block was 10 cm above the surface of the platform. Mice were given two blocks of four trials on four consecutive days to find the visible platform.

Histology

For the staining of ATXN1 nuclear aggregates in Purkinje neurons, cerebellar tissue was collected from 37-week old mice and drop fixed in 10% formalin for 24 hours. Standard techniques were used to embed tissue in paraffin and to take coronal sections 10 μm thick in serial section every 100 μm. Sections were then prepared for staining by being deparaffinized and rehydrated. Slides were treated with antigen retrieval by boiling for 9 min in 10 mM sodium citrate, 0.05%

Tween 20, pH 6.0. anti-ATXN1 11NQ rabbit polyclonal antibody (1) was incubated at a dilution of 1:2000 for approximately 16 h at 4°C. This primary antibody was then detected with biotinylated horse anti-rabbit IgG (Vector Laboratories; 1:1000) and visualized using an ABC reagent kit (Vector Laboratories) according to the manufacturer's recommendations. Brightfield images were acquired using an ECHO Revolve microscope. All immunohistochemistry experiments were performed with 4-6 mice per genotypes and figures show representative results. Percent of Purkinje neurons with aggregates per mouse was determined by combining 5 fields of view at 40x and counting total number of Purkinje neurons together with the number of Purkinje neurons with nuclear ATXN1 aggregates.

Supplemental References

1. Lasagna-Reeves CA et al. A native interactor scaffolds and stabilizes toxic Ataxin-1 oligomers in SCA1. *Elife* 2015;4(MAY). doi:10.7554/eLife.07558
